# RNA Sequencing Identifies New RNase III Cleavage Sites in *Escherichia coli* and Reveals Increased Regulation of mRNA

**DOI:** 10.1128/mBio.00128-17

**Published:** 2017-03-28

**Authors:** Gina C. Gordon, Jeffrey C. Cameron, Brian F. Pfleger

**Affiliations:** a Department of Chemical and Biological Engineering, University of Wisconsin—Madison, Madison, Wisconsin, USA; b Microbiology Doctoral Training Program, University of Wisconsin—Madison, Madison, Wisconsin, USA; University of Delaware

**Keywords:** *Escherichia coli*, RNA degradation, RNA sequencing, RNA stability, RNase III

## Abstract

Ribonucleases facilitate rapid turnover of RNA, providing cells with another mechanism to adjust transcript and protein levels in response to environmental conditions. While many examples have been documented, a comprehensive list of RNase targets is not available. To address this knowledge gap, we compared levels of RNA sequencing coverage of *Escherichia coli* and a corresponding RNase III mutant to expand the list of known RNase III targets. RNase III is a widespread endoribonuclease that binds and cleaves double-stranded RNA in many critical transcripts. RNase III cleavage at novel sites found in *aceEF*, *proP*, *tnaC*, *dctA*, *pheM*, *sdhC*, *yhhQ*, *glpT*, *aceK*, and *gluQ* accelerated RNA decay, consistent with previously described targets wherein RNase III cleavage initiates rapid degradation of secondary messages by other RNases. In contrast, cleavage at three novel sites in the *ahpF*, *pflB*, and *yajQ* transcripts led to stabilized secondary transcripts. Two other novel sites in *hisL* and *pheM* overlapped with transcriptional attenuators that likely serve to ensure turnover of these highly structured RNAs. Many of the new RNase III target sites are located on transcripts encoding metabolic enzymes. For instance, two novel RNase III sites are located within transcripts encoding enzymes near a key metabolic node connecting glycolysis and the tricarboxylic acid (TCA) cycle. Pyruvate dehydrogenase activity was increased in an *rnc* deletion mutant compared to the wild-type (WT) strain in early stationary phase, confirming the novel link between RNA turnover and regulation of pathway activity. Identification of these novel sites suggests that mRNA turnover may be an underappreciated mode of regulating metabolism.

## IMPORTANCE

The concerted action and overlapping functions of endoribonucleases, exoribonucleases, and RNA processing enzymes complicate the study of global RNA turnover and recycling of specific transcripts. More information about RNase specificity and activity is needed to make predictions of transcript half-life and to design synthetic transcripts with optimal stability. RNase III does not have a conserved target sequence but instead recognizes RNA secondary structure. Prior to this study, only a few RNase III target sites in *E. coli* were known, so we used RNA sequencing to provide a more comprehensive list of cleavage sites and to examine the impact of RNase III on transcript degradation. With this added information on how RNase III participates in transcript regulation and recycling, a more complete picture of RNA turnover can be developed for *E. coli*. Similar approaches could be used to augment our understanding of RNA turnover in other bacteria.

## INTRODUCTION

RNases are best known for their role in processing stable RNAs (e.g., rRNA, tRNA) to their mature, functional forms. In addition, RNases are critical for the inactivation and recycling of protein-encoding mRNA transcripts. In *Escherichia coli* and *Bacillus subtilis*, the best-studied models of bacterial RNA turnover, endonucleases and exonucleases act in concert, often facilitated by formation of a multienzyme complex, termed a degradosome, to inactivate and recycle transcripts to single nucleotides (1, 2). A common, generic model of mRNA turnover begins with a rate-determining, inactivating cleavage by an endoribonuclease followed by exoribonuclease-mediated turnover of secondary transcript fragments (3). That said, specific mechanisms of mRNA decay are known for a limited number of transcripts and predictions of how an unstudied transcript will decay are not available. This knowledge gap is due to the experimental complexity created by the presence of redundant decay pathways, the interaction of multiple enzyme classes, and the strong influence of environmental factors on RNase activity in each case. Furthermore, RNase activity can be blocked by specific protein inhibitors (4, 5), impaired by the presence of RNA secondary structures, or accelerated by processing of other RNases. In addition, the substrate preference (i.e., sequence, structure) of endonucleases is poorly understood. RNases can demonstrate highly selective cleavage specificity *in vivo* but often significant nonspecific activity *in vitro* (6). Our imprecise understanding of the factors that influence RNase activity prevents prediction of preferred cleavage sites, transcript abundance, and RNA half-life (*t*_1/2_) from genomic sequence. This report begins to address this gap by cataloging the native RNA targets of RNase III in *E. coli*.

RNase III is a widely distributed endoribonuclease that plays many roles in processing bacterial and viral RNAs, and yet a complete catalog of its targets and regulatory effects remains unavailable. RNase III acts as a homodimer with two Mg^2+^-dependent active sites that cleave double-stranded RNA (dsRNA), leaving a 5′ phosphate and 3′ hydroxyl with a 2-nucleotide (nt) overhang (7). RNase III is best known for its role in processing rRNA to release mature 16S and 23S rRNAs (8–11). RNase III is essential in *B. subtilis* due to its role in cleaving the duplex of two RNAs encoding toxins on two prophages (12). In *E. coli*, the RNase III gene (*rnc*) regulates its own expression by binding and cleaving a secondary structure in the 5′ untranslated region (5′ UTR), causing rapid degradation of the *rnc* message (13). The negative autoregulation is possible because RNase III accounts for only 0.01% of the total protein (14) and can be effectively titrated away from its own message by abundant targets such as rRNA. Another well-known example of RNase III regulation of mRNA is *pnp*, which encodes PNPase, a 5′–3′ exoribonuclease. RNase III cleaves a secondary structure in the 5′ UTR, leading to rapid degradation of the *pnp* message (15). RNase III can also affect protein expression through a translational control mechanism where the ribosome binding site (RBS) is occluded in RNA secondary structure until RNase III cleavage makes it accessible (e.g., native *E. coli* alcohol dehydrogenase [*adhE*]; cIII in lambda phage [16, 17]). There are also many examples of RNase III cleavage that alter RNA maturation and the abundance of RNA duplexes. RNase III targets several locations in bacteriophage lambda (17–19), T7 phage (20), and T3 phage (21). In addition to maturation of viral RNAs, RNase III also participates in the bacterial immune system (type II clustered regularly interspaced short palindromic repeat [CRISPR]/Cas systems) by releasing mature guide RNAs from CRISPR-encoded arrays (22). Cleavage of antisense RNA by RNase III can control plasmid copy number (23). Eukaryotic RNase III family members (Dicer and Drosha) have been shown to be central players in RNA interference and microRNA processing (24, 25). Beyond RNA cleavage, RNase III can alter secondary structure through binding to double-stranded RNA without cleaving (26). With such an expansive list of viral and cellular RNA targets, we wondered if other native mRNAs were subject to RNase III cleavage and how cleavage impacted transcript half-life.

In this study, we performed RNA sequencing (RNA-seq) on an *E. coli* model system to confirm known sites, identify novel targets, and determine the impact of cleavage events on transcript degradation and metabolic phenotypes. To find cleavage sites, we compared the abundances of sequencing reads across the transcriptome of a wild-type (WT) *E. coli* strain and an *rnc* deletion mutant. The RNA sequencing approach provided wider coverage and unprecedented resolution of mRNA abundance at each position in the transcriptome compared to prior studies that used quantitative PCR (qPCR), Northern blotting, or microarrays (27). In addition to data collected from exponentially growing cells, we observed the effects of RNase III cleavage on transcript degradation by collecting samples in a time course after stopping nascent transcription with rifampin. Combined, our datasets allowed us to pinpoint novel RNase III cleavage sites located within transcripts encoding important metabolic enzymes, near alternative promoters, and within transcription attenuators.

## RESULTS

### Determination of global RNase III targets.

To study RNase III processing of the *E. coli* transcriptome, we collected total RNA from two strains of exponentially growing *E. coli* strains, MG1693 (WT) and SK4455 (*rnc* deletion mutant). Samples were harvested over a 20-min time course following addition of rifampin to each culture. Ribosomal RNA was reduced from these samples, and the resulting pool was processed for RNA sequencing. The resulting sequencing reads were mapped to the *E. coli* genome and quantified per base using Integrated Genomics Viewer (IGV) tools (28).

**FIG 1:**
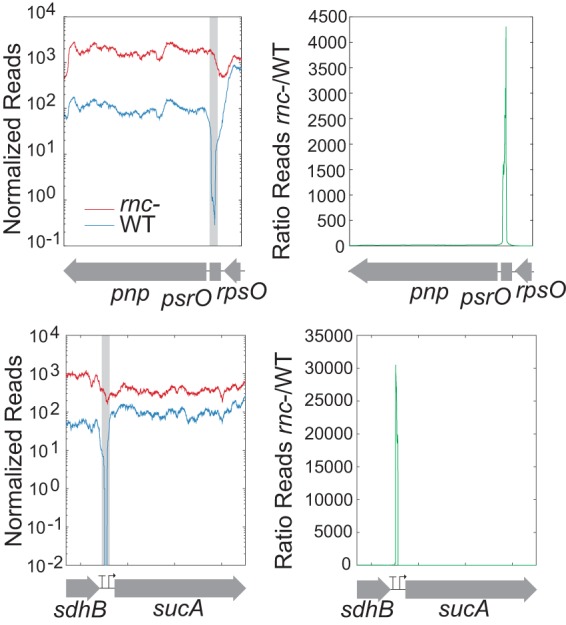
Identification of RNase III cleavage sites by comparison of the levels of RNA sequencing coverage between the wild-type strain (WT) and the RNase III mutant (*rnc*-). Read coverage across genomic locations of known cleavage sites near *sucA* and *pnp* are shown as well as the location of the known cleavage site (gray box). Novel cleavage sites were identified by looking for peaks in the ratio of reads between the *rnc* deletion mutant and the WT.

**FIG 2:**
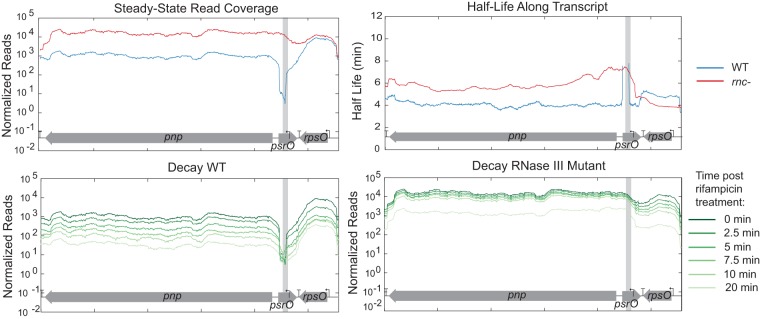
RNA-seq coverage around known RNase III cleavage site 5′ of *pnp*. Degradation throughout time is shown both for the WT and *rnc* deletion mutant, with time zero minutes shown in dark green and lighter green at 20 min. Half-lives in both the WT strain and *rnc* deletion mutant were calculated and displayed only when the *R*^2^ value was ≥0.7. The cleavage site is identified with a gray box.

We examined two well-known RNase III processing sites, 5′ of *pnp* and *sucA* (Fig. 1), to identify hallmarks of cleavage in our RNA-seq data. Prior to rifampin addition, WT samples (blue) exhibited drastically reduced read coverage compared to the read coverage of the *rnc* deletion mutant (red) at processing sites. The difference led to dramatic spikes in the ratio of *rnc* deletion mutant reads/WT reads near the site of RNase III cleavage (green). Beyond the cleavage site, the time course post-rifampin addition demonstrated a degradation pattern of the *pnp* transcript consistent with previous descriptions (29); RNase III cleavage results in a faster decay of the downstream secondary transcript (Fig. 2). In *rnc* deletion mutant samples, the downstream transcript is more stable, with a half-life of ~6 min compared to ~4 min in the WT. Previous studies using S1 nuclease mapping reported that the half-life of the *pnp* transcript was 8 min in the *rnc* deletion mutant and 1.5 min in the WT (30); others have reported a half-life of >40 min in the *rnc* deletion mutant (31). The RNA profiles of several other known RNase III targets were similarly changed (see Fig. S1 in the supplemental material).

10.1128/mBio.00128-17.1 FIG S1  Steady-state read coverage and decay throughout time in the WT strain and *rnc* deletion mutant for known RNase III processing sites (a) *corA* (71), (b) *mltD* (42), (c) *metY* (46), (d) *arfA* (41), (e) *rplL* (65), (f) *sucA* (64), (g) *tufB* (66), (h) *dicF* (67), (i) *proU* (68), (j) *bdm* (69), (k) *rng* (63), (l) *betT* (70), (m) *nirB* (27), and (n) an rRNA operon (9, 10). A gray box marks the known cleavage site. Download FIG S1, PDF file, 2.2 MB. Copyright © 2017 Gordon et al. 2017 Gordon et al. This content is distributed under the terms of the Creative Commons Attribution 4.0 International license.

On the basis of these observations, we devised a search algorithm for identifying putative RNase III processing sites. Specifically, we plotted the ratio of reads from the *rnc* deletion mutant to reads from the WT and searched for sequences where the *rnc* deletion mutant strain was dramatically more abundant than the WT in short windows—i.e., sites where cleavage would reduce RNA abundance in the WT. From this data set, we created a list of potential cleavage sites that had a ratio of reads between the *rnc* deletion mutant and WT of 30 in both replicates. This ratio was chosen because this was the lowest ratio that we observed for a known site, *tufB*. With this criterion, there were numerous locations selected that corresponded to shoulders of potential sites, so we required the sites to be at least 400 bp apart. The list comprised the majority of known processing sites as well as novel putative cleavage sites (see Table S1 in the supplemental material). The top 20 novel cleavage sites as well as the genes that are affected by the cleavage event are listed in Table 1. Sites were widely distributed across the genome, falling both within coding regions and within untranslated regions.

10.1128/mBio.00128-17.8 TABLE S1  Putative RNase III cleavage sites as identified with RNA-seq data. Download TABLE S1, XLSX file, 0.1 MB. Copyright © 2017 Gordon et al. 2017 Gordon et al. This content is distributed under the terms of the Creative Commons Attribution 4.0 International license.

**TABLE 1:** Known RNase III cleavage sites and top putative RNase III cleavage sites as identified by RNA-seq coverage differences between the WT strain and *rnc* deletion mutant^a^

Location	Annotation	Ratio 1	Ratio 2	WT reads Rep. A (no.)	WT reads Rep. B (no.)	ΔRNase III reads (no.)	Gene(s) affected	Reference or result of *in vitro* cleavage assay
Known cleavage sites identified in this study								
4166537	Intergenic (*muri*-*rrsB*)	415	587	41	29	17,025	rRNA (*rrsB*)	10
4035410	Intergenic (*hemG*-*rrsA*)	314	925	53	18	16,647	rRNA (*rrsA*)	10
3311286	*psrO*	3,782	3,782	3	3	11,347	*pnp*	15
2731278	Intergenic (*rrsG*-*clpB*)	487	534	23	21	11,206	rRNA (*rrsG*)	10
223653	Intergenic (*gmhB*-*rrsH*)	271	491	29	16	7,853	rRNA (*rrsH*)	10
3428875	Intergenic (*rrsD*-*yrdA*)	340	234	20	29	6,791	rRNA (*rrsD*)	10
3943398	Intergenic (*rrsC*-*gltU*)	1,309	2,619	4	2	5,237	rRNA (*rrsC*)	10
4209733	Intergenic (*rrsE*-*gltV*)	4,959	2,480	1	2	4,959	rRNA (*rrsE*)	10
4168221	Intergenic (*rrsB*-*gltT*)	123,700	123,700	0.01	0.01	1,237	rRNA (*rrsB*)	10
4168637	Intergenic (*gltT*-*rrlB*)	120	419	7	2	837	rRNA (*rrlB*)	9
3427176	Intergenic (*Ileu*-*rrsD*)	77,500	775	0.01	1	775	rRNA (*rrsD*)	10
3397879	*yhdE*	114	98	6	7	684	*rng*	63
225355	Intergenic (*rrsH*-*ileV*)	64,300	643	0.01	1	643	rRNA (*rrsH*)	10
4037115	Intergenic (*rrsA*-*ileT*)	62,600	62,600	0.01	0.01	626	rRNA (*rrsA*)	10
3318176	Intergenic (*rimP*-*metY*)	102	609	6	1	609	*metY*	46
4037523	*rrlA*	149	223	3	2	446	rRNA (*rrlA*)	9
758515	P67/*sucA*	30,500	305	0.01	1	305	*sucA*	64
4207874	Intergenic (*purH*-*rrsE*)	29,000	290	0.01	1	290	rRNA (*rrsE*)	10
3946623	Intergenic (*rrlC*-*rrfC*)	22,700	227	0.01	1	227	rRNA (*rrlC*)	9
2729186	Intergenic (*rrlG*-*gltW*)	55	221	4	1	221	rRNA (*rrlG*)	9
4040443	Intergenic (*rrlA*-*rrfA*)	20,300	203	0.01	1	203	rRNA (*rrlA*)	9
4212954	Intergenic (*rrlE*-*rrfE*)	19,500	65	0.01	3	195	rRNA (*rrlE*)	9
3941526	Intergenic (*yieP*-*rrsC*)	16,200	41	0.01	4	162	rRNA (*rrsC*)	10
4171559	Intergenic (*rrlB*-*rrfB*)	12,100	12,100	0.01	0.01	121	rRNA (*rrlB*)	9
3423873	Intergenic (*rrfD*-*rrlD*)	11,700	117	0.01	1	117	rRNA (*rrlD*)	9
2726273	Intergenic (*rrfG*-*rrlG*)	11,500	58	0.01	2	115	rRNA (*rrlG*)	9
Putative cleavage sites identified in this study								
122961	Intergenic (*pdhR*-*aceE*)	6,977	1,550	2	9	13,954	*aceE*, *aceF*, *lpdA*	Y
639695	Intergenic (*ahpC*-*ahpF*)	900	900	4	4	3,598	*ahpC*	Y
1799278	Intergenic (*pheM*-*rplT*)	639	799	5	4	3,196	*pheM*, *pheS*, *pheT*	Y
4330944	*proP*	66	83	15	12	992	*prop*	Y
3889207	tnaA*	301	129	3	7	903	*tnaC*, *tnaA*, *tnaB*	Y
755579	sdhD*	545	42	1	13	545	*sdhC*, *sdhD*	Y
3683389	*dctA*	74	74	7	7	518	*dctA*	Y
2171807	Intergenic (*gatR*-*gatD*)	213	47	2	9	426	*gatR*, *gatD*	N
3609652	*yhhQ*	58	77	4	3	231	*yhhQ*	N
951147	Intergenic (*pflA*-*pflB*)	15,800	15,800	0.01	0.01	158	*pflB*	Y
2351931	*glpT*	14,300	143	0.01	1	143	*glpT*	N
2237178	mglC*	133	13,300	1	0.01	133	*mglC*, *mglA*, *mglB*	N
4307817	alsE*	126	63	1	2	126	*alsB*, *Alsa*, *alsE*, *alsC*, *alsK*	N
2090031	hisL	11,900	60	0.01	2	119	*hisL*	Y
1166043	Intergenic (*ycfP*-*ndh*)	10,800	10,800	0.01	0.01	108	*ndh*	Y
159578	gluQ*	10,400	52	0.01	2	104	*gluQ*	N
444573	Intergenic (*panE*-*yajQ*)	48	32	2	3	96	*yajQ*, *panE*	Y
1622816	Intergenic (*yneM*-*mgrR*)	9,100	91	0.01	1	91	*yneM*, *mgrR*	N

^a^ Positions with zero reads were given a pseudocount of 0.01 reads. Asterisks (*) indicate that multiple cleavage sites were identified within a region and combined into one entry in the table. “Y” indicates there was observed cleavage of an *in vitro* transcribed transcript when incubated with purified enzyme, while “N” indicates a lack of observed cleavage. Ratio 1, number of reads in ΔRNase III divided by number of reads in WT replicate A; Ratio 2, number of reads in ΔRNase III divided by number of reads in WT replicate B; Rep. A, number of reads in WT replicate A; Rep. B, number of reads in WT replicate B.

### Verification of novel RNase III sites with *in vitro* cleavage assays.

**FIG 3:**
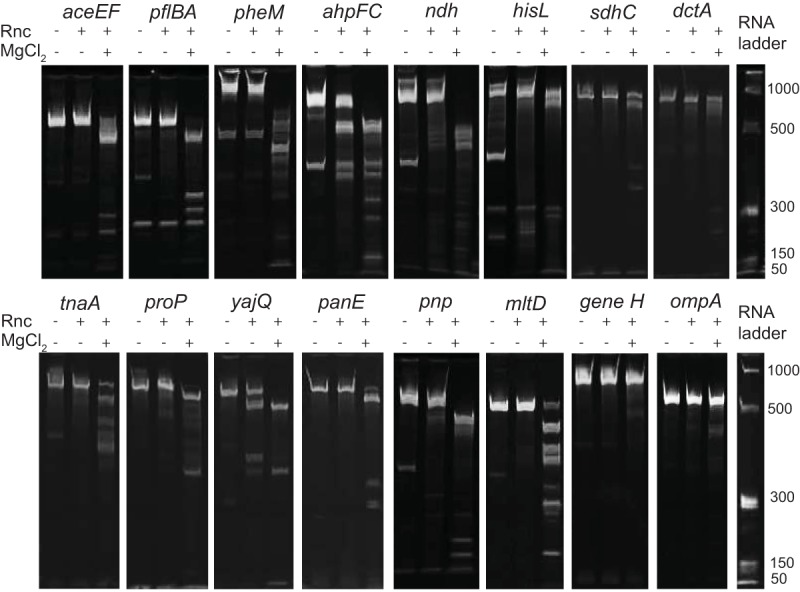
RNase III cleavage of *in vitro* transcripts. Five micrograms of *in vitro* transcribed RNA was incubated with purified RNase III and activated with MgCl_2_ at 37°C for 15 min. Five hundred nanograms of RNA was removed before the addition of enzyme and MgCl_2_. Samples were run on a denaturing polyacrylamide gel and imaged with ethidium bromide.

We performed *in vitro* cleavage assays to confirm the processing of 19 putative RNase III cleavage sites selected using the following criteria: (i) top ranking based on search algorithm; (ii) RNA-seq pattern resembling those of known cleavage sites; and (iii) predicted secondary structure containing a double-stranded region greater in size than the minimum RNase III target size of 22 nt. For *in vitro* tests, synthetic RNA templates were designed such that putative cleavage sites were positioned ~150 nucleotides from the 5′ end to ensure that the cleavage product was clearly visible when it was separated from uncut RNA on a denaturing 5% polyacrylamide gel. A T7 promoter was included in the 5′ end of the primer used to create PCR products for use as the templates in *in vitro* transcription reactions. RNA made from *in vitro* transcription was quantified and incubated with purified *E. coli* MG1655 RNase III. Reactions were activated by the addition of MgCl_2_ and incubated at 37°C for 15 min before quenching with excess EDTA. *In vitro* reaction products were separated on a 5% polyacrylamide urea gel. Using this approach, we demonstrated RNase III cleavage of 11 putative sites near *aceEF*, *pflBA*, *pheM*, *ahpFC*, *ndh*, *hisL*, *sdhC*, *dctA*, *tnaA*, *proP*, and *yajQ-panE* (Fig. 3). For each putative cleavage site, the band corresponding to the full-length transcript decreased in intensity or disappeared after addition of both enzyme and MgCl_2_. Analogously, new bands, shorter in length, appeared in each of these samples. Two known cleavage sites (*pnp* and *mltD*) were tested as well as two negative controls not known to be processed by RNase III (*gene H* from bacteriophage phi X174 and *ompA*, a highly structured RNA in *E. coli*). Bands corresponding to full-length RNA for both negative controls remained intact after 15 min of incubation with purified RNase III. In 8 of the 19 putative sites, bands corresponding to full-length transcript remained after 15 min (Fig. S2), suggesting that these sequences are not processed by RNase III *in vitro*. It is possible that these sites may be cleaved *in vivo* through interactions with other RNAs or altered secondary structure. RNase III cleavage of dsRNA consisting of antisense RNA and sense RNA has been shown to be prevalent and may play a major role in gene regulation (32). Unfortunately, we were unable to determine if this was the case for our putative cleavage sites because our RNA-seq data did not contain stranded information. The presence of an antisense transcript involved in RNase III cleavage could explain our inability to show *in vitro* cleavage of two putative cleavage sites, integenic *yneM*-*mgrR* and *yhhQ*, since both of these sites were previously shown to have RNase III-dependent antisense transcripts (32).

10.1128/mBio.00128-17.2 FIG S2  Lack of RNase III cleavage of *in vitro* transcripts. The top panel shows other leader sequences that were examined because we found RNase III processing of other leader transcripts. The bottom panel shows putative targets identified using the RNA-seq data. Five micrograms of *in vitro* transcribed RNA was incubated with purified RNase III and activated with MgCl_2_ at 37°C for 15 min. Five hundred nanograms of RNA was removed before the addition of enzyme and MgCl_2_. Samples were run on a denaturing polyacrylamide gel and stained with ethidium bromide and imaged. Download FIG S2, EPS file, 1.7 MB. Copyright © 2017 Gordon et al. 2017 Gordon et al. This content is distributed under the terms of the Creative Commons Attribution 4.0 International license.

**FIG 4:**
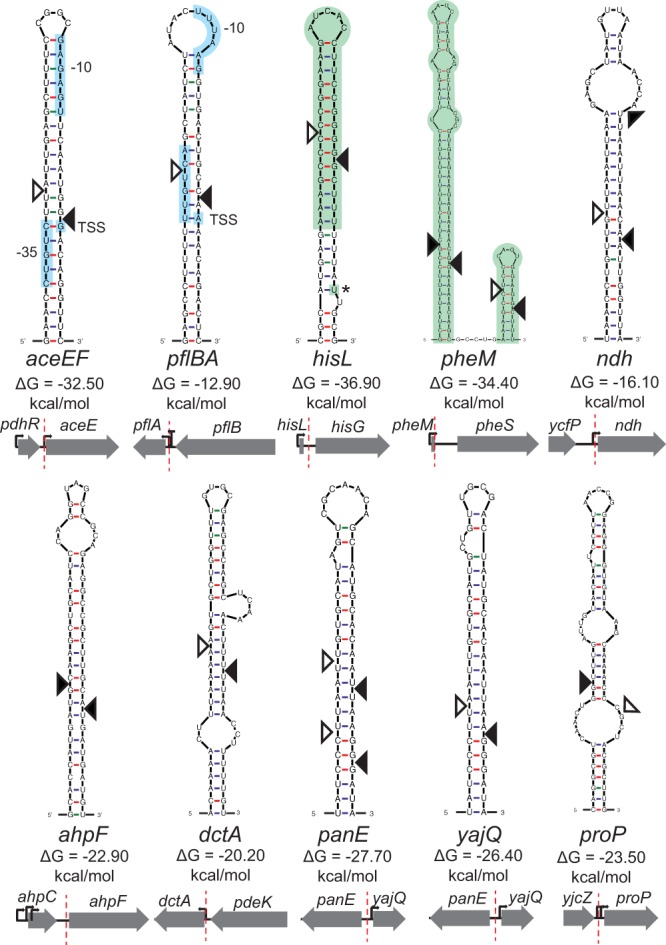
Predicted secondary structure of novel RNase III sites. Dark triangles indicate the cleavage site identified by 5′ RACE. Open triangles indicate the putative location of the corresponding cut required to leave 2-nt overhangs, characteristic of RNase III cleavage. Colored sections highlight other known regulatory elements such as promoters and structures involved in transcriptional attenuation. The orientation of surrounding genes and the location of an RNase III cleavage site are depicted below the structure.

Using Mfold (33), we were able to identify predicted long double-stranded regions for all 11 confirmed locations of the cleavage sites. We confirmed these locations by performing 5′ rapid amplification of cDNA ends (RACE) on RNA extracted from samples at the end of *in vitro* cleavage reactions (Fig. 4). Three sequences, near *pheM*, *ndh*, and *panE*, contained multiple cleavage sites that were individually identified by sequencing 5′ RACE fragments subcloned into pGEM. Several cleavage sites were found adjacent to or within other regulatory elements such as promoters (*aceEF* and *pflBA*) and attenuators (*hisL* and *pheM*). We noticed that three of the verified sites were close to leader peptides (*hisL*, *pheM*, and *tnaC*) and wondered if RNase III was involved in the turnover of other leader peptides. We performed RNase III cleavage assays on 10 other transcripts encoding leader peptides but saw that none were processed by RNase III *in vitro* (Fig. S2).

### RNase III cleavage impacts mRNA transcript degradation rates.

**FIG 5:**
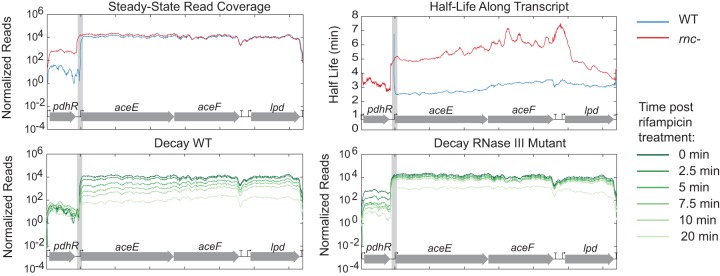
Read coverage and decay near novel RNase III cleavage site *aceEF* that shows increased degradation after RNase III cleavage and stabilization in the *rnc* deletion mutant. Normalized read coverage is displayed along the coordinates of the *E. coli* genome. Degradation throughout time is shown for both the WT and the *rnc* deletion mutant, with time zero minutes shown in dark green and lighter green at 20 min. Half-lives in both the WT strain and the *rnc* deletion mutant were calculated and displayed only when the *R*^2^ value was ≥0.7. The cleavage site is identified with a gray box.

The expansion of known RNase III cleavage sites allowed us to examine the effect of RNase III cleavage on transcript decay. Overall, our observations can be grouped into four mechanisms of regulation mediated by RNase III: (i) destabilization of the transcript; (ii) stabilization of the transcript; (iii) degradation of transcripts encoding leader peptides involved in transcriptional attenuation; and (iv) processing that mimics transcription from an alternate promoter. We found that in 10 of the 18 putative sites highlighted in Table 1, RNase III cleavage events led to destabilization of WT transcripts compared to *rnc* deletion mutant transcripts. For example, the half-life of the WT *aceEF* transcript was ~3 min and the half-life of this transcript in *rnc* deletion mutant was ~5 min (Fig. 5). This is the same as the pattern seen with known RNase III cleavage sites such as* pnp* (13), where half-lives were shorter in the WT than in the *rnc* deletion mutant. Other RNase III cleavage sites following this pattern included those near *tnaC*, *dctA*, *pheM*, *sdhC*, *yhhQ*, *glpT*, *aceK*, *gluQ*, and* proP* (Fig. S3A to I). One cleavage site that we identified, *proP*, has been previously described but only under conditions where *proP* was transcribed from the osmoregulated P1 promoter (34). Here, we identified this cleavage site in exponentially growing cells, highlighting that this cleavage event also occurs without the presence of osmotic stress. The steady-state *proP* transcript levels in this study agreed with what was previously described, showing an increase in abundance in the *rnc* mutant compared to the WT. Our data also support their conclusion that cleavage of the *proP* transcript by RNase III leads to degradation and a shorter half-life compared to uncut transcript.

10.1128/mBio.00128-17.3 FIG S3  Steady-state read coverage and decay throughout time in the WT strain and *rnc* deletion mutant for *tnaC*, *dctA*, *pheM*, *sdhC*, *yhhQ*, *glpT*, *aceK*, *gluQ*, *and proP* transcripts (a to i). All transcripts in the WT seemed to be degraded more quickly than in the *rnc* deletion mutant. We hypothesize that the half-lives could not be calculated for several transcripts in the WT because the degradation happened very rapidly. Download FIG S3, PDF file, 1.7 MB. Copyright © 2017 Gordon et al. 2017 Gordon et al. This content is distributed under the terms of the Creative Commons Attribution 4.0 International license.

**FIG 6:**
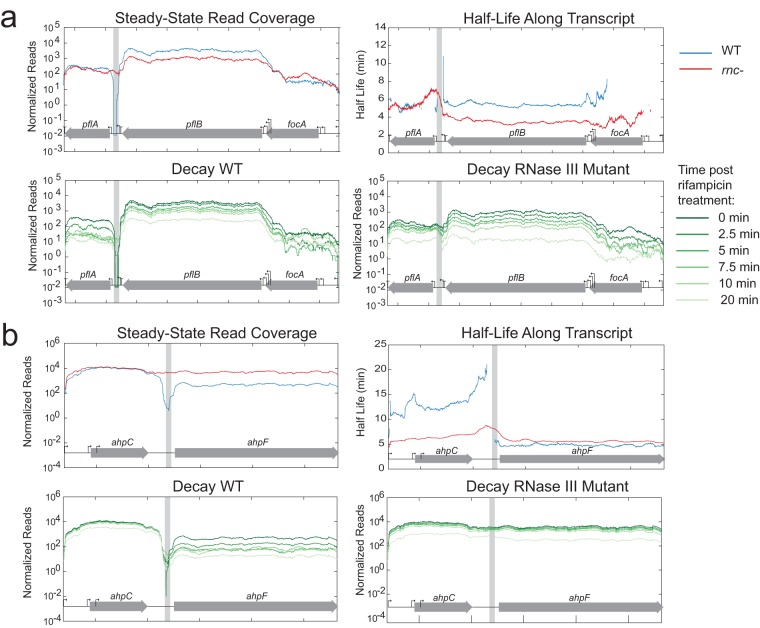
Steady-state read coverage and decay throughout time in the WT strain and *rnc* deletion mutant for *pflBA* (a) and *ahpFC* (b) transcripts that show increased stability in the WT.

In contrast, we also identified examples where RNase III processing had no impact on transcript stability (e.g., upstream of *ndh*; Fig. S4) or led to an increased half-life of the resulting secondary transcript. For example, we observed that RNase III cleavage at a site between *pflB* and *pflA* led to stabilization of the *pflB* transcript in the WT compared to *rnc* deletion mutant (~5.5 min for the WT and ~3.5 min for the *rnc* deletion mutant). The cleavage site is located very close to the promoter region of *pflA* (Fig. 6A). The transcription likely originated upstream, presumably from the promoter in front of *pflB*, and proceeded through a strong rho-independent terminator (35). The cleavage event at the 3′ end of the *pflB* transcript may serve to ensure the separation of the RNAs encoding *pflB* and *pflA* and may mimic the event seen with the transcript formed from the promoter preceding *pflA*. Similarly, cleavage at a site between *ahpC* and *ahpF* stabilizes the *ahpC* transcript (~14-min half-life in the WT strain and ~6-min half-life in *rnc* deletion mutant) (Fig. 6B) while not changing the stability of *ahpF*. Additionally, cleavage at a site in the 5′ UTR of *yajQ* stabilizes the transcript in the WT relative to the *rnc* deletion mutant (~6 min for the WT and ~3 min for the *rnc* deletion mutant) (Fig. S5). Only the stability of *yajQ* was affected, but it is possible that *panE* might be influenced by this cleavage event. Due to the complementary nature of these hairpins, very similar structures could form from transcript of the plus strand that encodes *yajQ* and the minus strand that encodes *panE* (Fig. 4).

10.1128/mBio.00128-17.4 FIG S4  Steady-state read coverage and decay throughout time in the WT strain and *rnc* deletion mutant for the *ndh* transcript. There was no difference in half-life between the WT strain and *rnc* deletion mutant. Download FIG S4, EPS file, 2.1 MB. Copyright © 2017 Gordon et al. 2017 Gordon et al. This content is distributed under the terms of the Creative Commons Attribution 4.0 International license.

10.1128/mBio.00128-17.5 FIG S5  Steady-state read coverage and decay throughout time in the WT strain and *rnc* deletion mutant for *yajQ* and *panE* transcripts. Due to the lack of stranded RNA-seq data, we were unable to determine if the dip in coverage was resulting from processing of the *yajQ* transcript on the positive strand or the *panE* transcript on the negative strand. There was a difference only in the half-life of the *yajQ* transcript. Download FIG S5, EPS file, 1.7 MB. Copyright © 2017 Gordon et al. 2017 Gordon et al. This content is distributed under the terms of the Creative Commons Attribution 4.0 International license.

### RNase III-mediated processing of transcripts encoding attenuation-sensing peptides.

**FIG 7:**
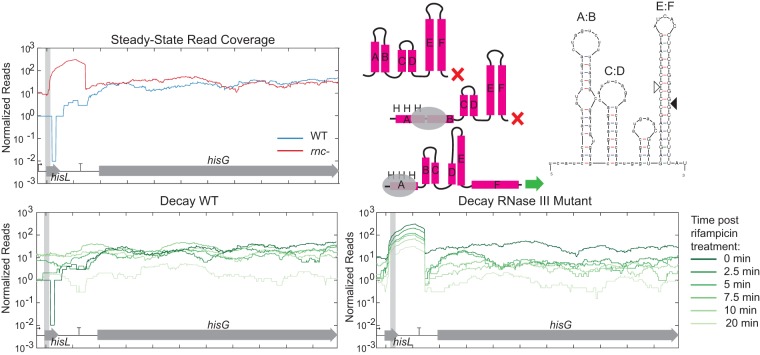
RNase III initiates decay of terminated *hisL* leader transcript. Steady-state read coverage and decay throughout time in the WT strain and *rnc* deletion mutant for *hisL* are shown in addition to the predicted secondary structures of the termination and attenuation structures. The verified RNase III cleavage site is indicated with a filled triangle.

**FIG 8:**
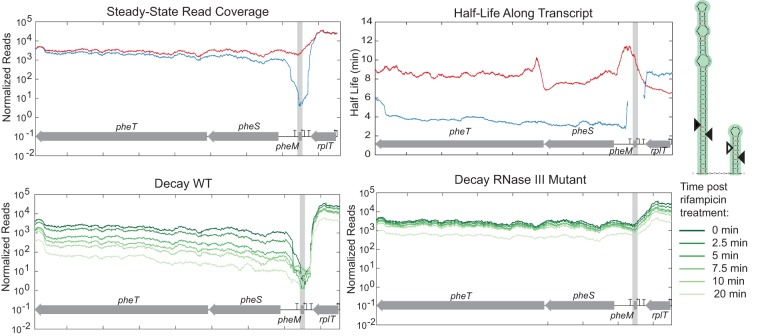
RNase III initiates decay of terminated *pheM* leader transcript. Steady-state read coverage and decay throughout time in the WT strain and *rnc* deletion mutant for *pheM* are shown in addition to the predicted secondary structure of the termination structure. The verified RNase III cleavage site is indicated with a filled triangle.

The placement of several RNase III cleavage sites may indicate additional levels of regulation. Two genes containing verified novel RNase III cleavage sites, *hisL* and *pheM*, are regulated by transcription attenuation. *hisL* and *pheM* encode short peptides with an abundance of histidine and phenylalanine, respectively (36, 37). There are a series of stem loops that partially overlap these peptides that can form two alternate structures; one leading to termination and the other leading to transcription of the downstream genes encoding histidine biosynthesis genes or phenylalanyl-tRNA synthetase genes. In the case of *hisL*, we observed an increased amount of this leader transcript in the *rnc* deletion mutant strain compared to the WT (Fig. 7). Using 5′ RACE on *in vitro* cleavage reactions, we found that this site overlapped with the E:F termination structure (38). An RNase III cleavage site was also identified in the *pheM* transcriptional attenuation structure (Fig. 8). Using 5′ RACE, we identified several different cleavage products with ends located within the termination structure. Unlike the *hisL* results, the leader structure did not accumulate in the *rnc* deletion mutant. Instead, degradation around this site resembled that of the well-known cleavage site 5′ of *pnp* (Fig. 2) where RNase III cleavage leads to degradation of the downstream message.

### RNase III activity affects a key metabolic node: pyruvate dehydrogenase.

**FIG 9:**
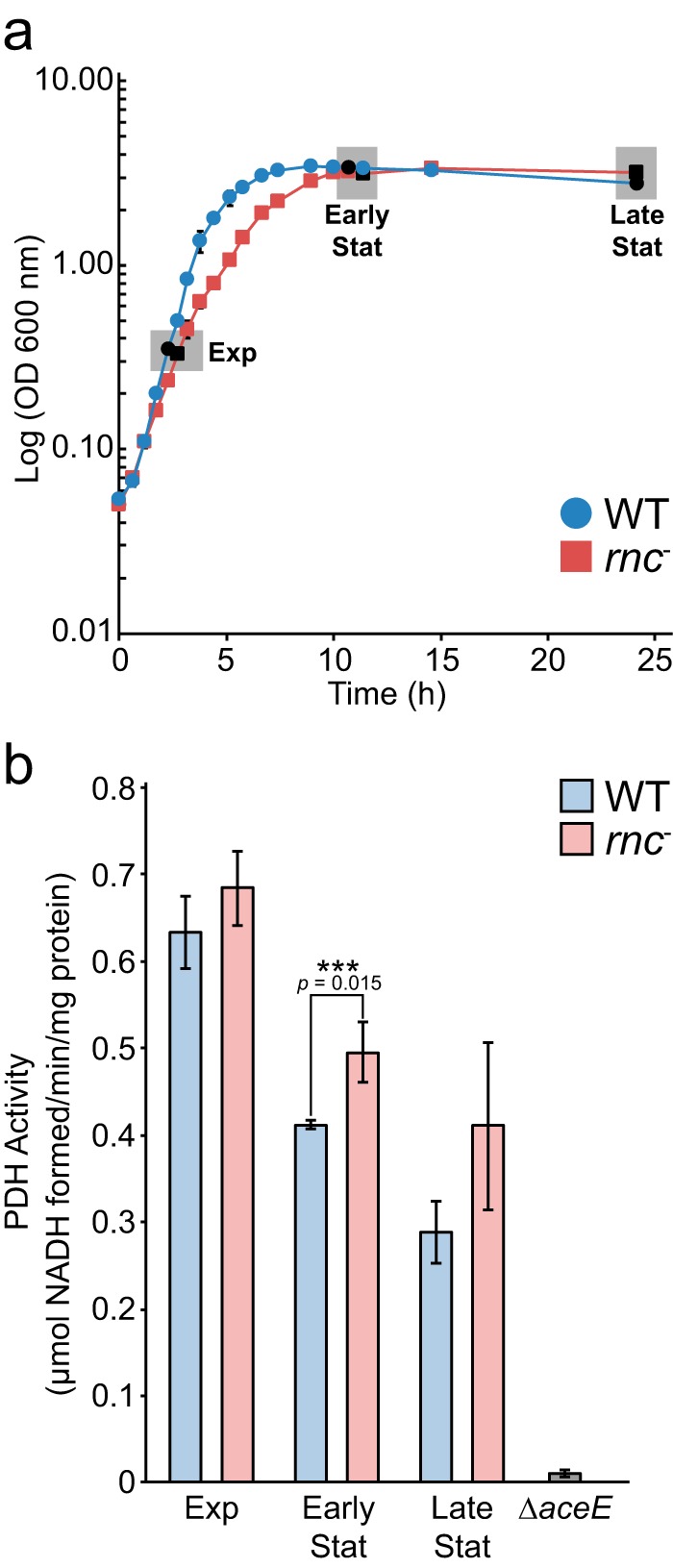
Pyruvate dehydrogenase activity in WT strain and *rnc* deletion mutant crude cell lysates. (a) Growth of MG1693 and *rnc* deletion mutant in LB medium supplemented with thymine (50 µg/ml). Cells were harvested during exponential (Exp), early stationary (Early Stat), and late stationary (Late Stat) growth for enzyme assays. (b) Pyruvate dehydrogenase (PDH) activity was measured spectroscopically as pyruvate-dependent NADH formation using lysates extracted from cells harvested at different growth phases. Lysates from a Δ*aceE* strain (JW0110-2; Keio Collection), grown to stationary phase in M9 minimal medium supplemented with glucose (0.2%) and acetate (0.1%), were used as a negative control because they lack PDH activity. Values represent means of results from three biological replicates, and error bars indicate ± standard deviations (SD) (***, *P* < 0.05 [Students *t* test, two-tailed]).

Many of the novel target sites identified in this study are linked to genes encoding metabolic enzymes. For instance, we observed a significant difference in the half-life values of the *aceE-aceF-lpd* transcript, encoding the multisubunit pyruvate dehydrogenase complex (PDH), between the WT and *rnc* deletion mutant strains. Given the critical role of pyruvate dehydrogenase, we examined the differences in PDH activity in the WT strain and *rnc* deletion mutant. Samples were taken from the WT and *rnc* deletion mutant grown in LB medium (with 50 μg/ml thymine) and M9 medium (with 50 μg/ml thymine, 0.4% glucose, and 0.2% Casamino Acids) during the exponential, early stationary, and late stationary phases. PDH activity was measured in a coupled enzymatic assay by monitoring pyruvate-dependent NADH formation in cell lysates. Increases in PDH activity were seen for the *rnc* deletion mutant during the early stationary phase but not during the exponential or late stationary phase (Fig. 9). RNase III processing of the *aceE-aceF-lpd* transcript could be responsible for altering PDH activity, presumably by altering the stability and abundance of the transcript (as shown in Fig. 5).

## DISCUSSION

### Mechanisms of RNase III-mediated regulation.

In addition to confirming known sites, our data identified 11 novel RNase III sites verified by *in vitro* cleavage assays. Together, the RNase III sites can be grouped into four mechanisms of regulation. First, cleavage by RNase III in the 5′ UTR can decrease mRNA abundance of protein-coding transcripts via a decrease in the half-life of the downstream message. Previously known examples of this phenomenon include *rnc*, *pnp*, and *metY*. Here, we found similar degradation patterns near* aceEF*, *proP*, *tnaC*, *dctA*, *pheM*, *hisL*, *sdhC*, *yhhQ*, *glpT*, *aceK*, and *gluQ*. For several of these transcripts (*proP*, *dctA*, *tnaC*, *sdhC*, *yhhQ*, *glpT*, *aceK*, and *gluQ*), we hypothesize that the degradation was rapid because there was no decrease in read coverage throughout the time course and the transcript abundance was low. We suspect that rapid decay occurred during the 3.5 min between rifampin addition and the initial sampling. Rapid transcript degradation following RNase III cleavage has been explained by the loss of *cis*-acting stabilizing elements—e.g., hairpins at the 5′ end of transcripts blocking RNase E activity (39) or hairpins at the 3′ end of transcripts blocking exonucleases polynucleotide phosphorylase (*pnp*) and RNase II (40). Alternatively, RNase III can inactivate transcripts by cleaving within coding regions (e.g., *nirB* [27], *arfA* [41], and *mltD* [42]). In this study, we found no verified novel sites within coding regions.

Second, RNase III can lead to increased mRNA abundance and/or expression of target genes. For instance, RNase III cleavage removes occlusion of the RBS controlling expression of alcohol dehydrogenase (*adhE*) (16). In the case of *ahpF*, *pflB*, and *yajQ*, novel sites identified in this study, there is an increase in stability of the processed transcript compared to the unprocessed transcript. We propose that these cleavage events leave stabilizing hairpins that protect secondary transcripts from degradation. Increased stability of RNase III-cleaved transcripts could be due to creation or placement of new stabilizing structures that impede further RNA turnover—e.g., placement of hairpins 3′ of *pflB* and *ahpC* to block exonucleases and 5′ of *yajO* that could slow RNase E processing (see Fig. S6 in the supplemental material). The importance of this novel mode of regulation is highlighted by the functions of the encoded proteins. AhpC and AhpF are located in the same operon and encode the subunits of alkyl hydroperoxide reductase, where AhpC forms a decameric ring and interacts with an AhpF dimer (43). Proper regulation and stoichiometry of these two genes transcribed in a single operon may be critical because they are among the most highly abundant proteins in the cell (44, 45). Another transcript that showed increased stability upon RNase III cleavage was pyruvate formate lyase (PflB), which is central to anaerobic metabolism. Together, these examples show that RNase III cleavage events can lead to both positive and negative regulation of gene expression.

10.1128/mBio.00128-17.6 FIG S6  Predicted secondary structure of transcripts following RNase III cleavage. Cleavage events at the 3′ end of the *pflB* and *ahpC* transcripts favor the formation of long hairpins at the 3′ end (Mfold [33]). Cleavage at the 5′ end of the *yajQ* transcript results in a transcript with a predicted hairpin at the 5′ end. Download FIG S6, EPS file, 1.7 MB. Copyright © 2017 Gordon et al. 2017 Gordon et al. This content is distributed under the terms of the Creative Commons Attribution 4.0 International license.

Third, we observed two novel cleavage sites within transcripts known to be involved in transcriptional attenuation (*hisL* and *pheM*). Here, formation of an RNA secondary structure leads to termination of the leader transcript that would otherwise read through the terminator and transcribe downstream genes. We pinpointed the *hisL* and *pheM* cleavage sites to these known secondary structures. Therefore, we hypothesize that after the attenuation mechanism terminates the leader transcript or continues transcription based on the concentration of charged tRNAs, RNase III is involved in recycling the terminated leader transcript. We observed a buildup of the *hisL* leader transcript in the RNase III mutant (Fig. 7) but not the *pheM* leader transcripts (Fig. 8). Instead, the *pheM* transcript resembled that of other cleavage sites where the downstream transcript decayed more rapidly. We hypothesize that RNase III does help turn over *pheM* leader transcripts, but under the growth conditions in which cells were harvested for RNA-seq, the major transcript was the read-through and not the attenuated transcript.

Fourth, we observed cleavage sites in several untranslated regions that produce products that are nearly identical to those produced by an alternative promoter. There is one published example of RNase III cleaving the *metY-nusA-infB* resulting in the downstream transcript resembling a transcript that originated from the internal P2 promoter (46). Two of our verified novel RNase III cleavage sites, near *aceEF* and *pflBA*, are also located very close to promoters. Both of the predicted hairpins contain the −35 site, −10 site, and transcription start site (TSS). These hairpin structures would form only from a transcript that began before the previous gene. In the case of *aceEF*, this cleavage event results in a downstream transcript that is identical to the mapped TSS of this promoter (47). RNase III cleavage of the *pflBA* transcript leads to the downstream transcript having a single additional “A” at the 5′ end compared to a transcript that would have originated from the promoter preceding *pflA* (48). It is possible that these previously described promoters identified by 5′ end mapping were actually the result of an RNase III processing event instead of the activity of a true promoter, but sequence elements with high similarity to σ^70^ promoters support the conclusion that these are true promoters.

We compared these novel RNase III cleavage sites to the consensus sequence (49) by identifying the proximal, middle, and distal boxes for the sites where we determined the cleavage site. None of these sequences perfectly matched the consensus sequence, but the *aceEF* site matched all but the third position of the proximal box (U-A instead of A-U). All other structures contained some similarities to the consensus sequence, but no striking pattern was observed. It remains to be seen whether the double-stranded structure is the more important recognition factor facilitating RNase III cleavage.

### RNase III alters metabolic activity.

RNase III-mediated regulation can have a significant impact on metabolism. Here, we observed and verified a cleavage site upstream of *aceE*, *aceF*, and *lpd*, genes encoding pyruvate dehydrogenase (PDH), and a site upstream of *pflB*, encoding pyruvate formate lyase. We observed an increase in the *aceEF-lpd* transcript levels in the RNase III mutant (Fig. 5) and observed that RNase III cleavage led to a reduction in PDH activity only during early stationary phase and not exponential or late stationary phase. These data are in agreement with a recent quantitative proteomics study that reported that an increase in growth rate led to increased AceE levels (50). Based on the lower growth rate of the *rnc* deletion mutant (Fig. 9a), a reduction in AceE levels might be expected, but we observed instead an increase in activity, transcript levels, and transcript half-life. Additionally, transcript levels of *aceE* have been shown to decrease significantly upon entry into stationary phase (51), and even if this reduction occurs in both the WT and the *rnc* deletion mutant, the observed increase in the half-life of *aceEF-lpd* (~5 min compared to ~3 min) could explain the observed increase in PDH activity in early stationary phase in the *rnc* deletion mutant. The effect of these processing events on transcripts encoding metabolic enzymes is complex because, although RNase III is known to be constitutively expressed (and autoregulates its own mRNA decay [29]), its activity has been shown to be affected by a protein inhibitor as well as by other, unknown factors (5).

**FIG 10:**
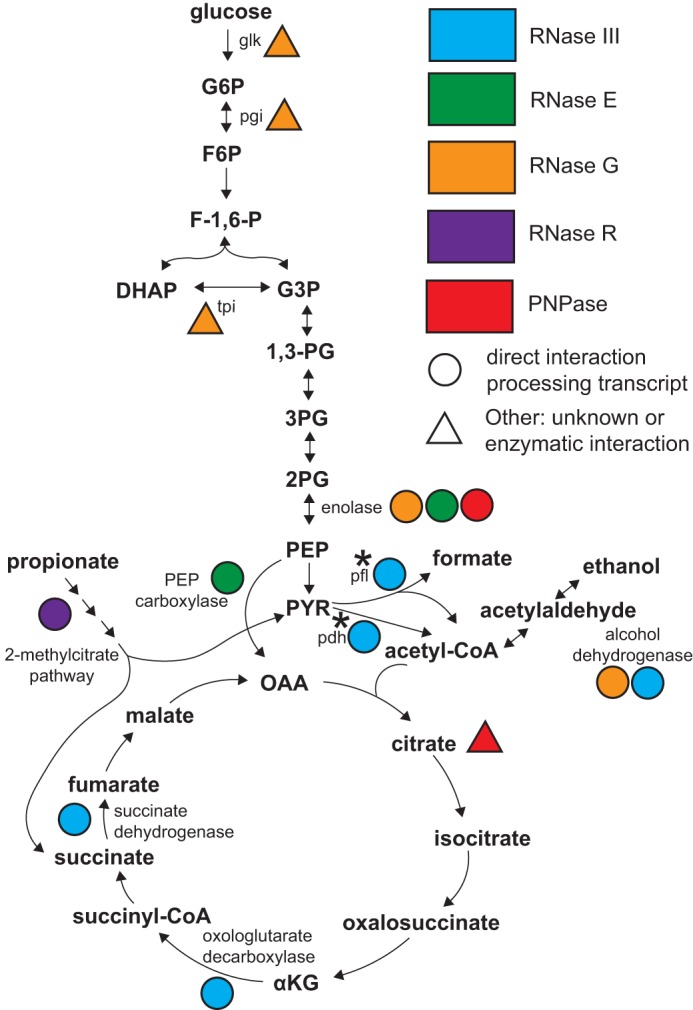
Influence of RNase activity on central metabolism. Circles indicate that the correspondingly colored RNase acts directly on the transcript encoding that enzyme (blue, RNase III; green, RNase E; orange, RNase G; purple, RNase R; red, PNPase). Colored triangles indicate that a particular RNase influences the abundance and activity of the enzyme via an unknown or indirect method. Triangles near metabolic intermediates indicate interactions of metabolites with RNases that influence RNase activity. A star indicates regulation identified in this study. Abbreviations: pgi, phosphoglucose isomerase; tpi, triosephosphate isomerase; pfl, pyruvate formate lyase; pdh, pyruvate dehydrogenase; G6P, glucose 6-phosphate; F6P, fructose 6-phosphate; F-1,6-P, fructose 1,6-bisphosphate; DHAP, dihydroxyacetone phosphate; G3P, glyceraldehyde 3-phosphate; 1,3-PG, 1,3-bisphosphoglycerate; 3PG, 3-phosphoglycerate; 2PG, 2-phosphoglycerate; PEP, phosphoenolpyruvate; PYR, pyruvate; αKG, alpha-ketoglutarate.

These new findings complement prior work that demonstrated that RNase III cleavage affected expression of central metabolic enzymes, including alcohol dehydrogenase (*adhE*), oxoglutarate dehydrogenase, and succinate dehydrogenase (*sdhCDAB*-*sucABCD*). This connection between ribonucleases and metabolic activity is not unprecedented. RNase E (*rne*) deletion mutants were unable to grow on M9 media with glucose without yeast extract or a mixture of amino acids, which indicated a defect in the connection between the glycolytic and tricarboxylic acid (TCA) pathways in *rne* deletion mutants (52). This was explained by a drastic decrease in phosphoenolpyruvate carboxylase production. We observed a similar growth defect when the *rnc* deletion mutant was grown in M9 media with glucose, but this growth defect could be rescued by adding 0.2% Casamino Acids (data not shown). Both RNase E and RNase III are involved in the regulation of enzymes that connect glycolysis and the TCA cycle (52) (Fig. 10). In addition to these two examples, there are a significant number of examples of how ribonucleases regulate metabolic enzymes via RNA stability and other mechanisms. RNase R has been shown to hydrolyze the *prpBCDE* operon encoding enzymes in the 2-methylcitrate pathway enabling propionate degradation under oxic but not anoxic conditions (53). Additional support for the link between RNases and central metabolism includes the presence of the degradosome where the key glycolytic enzyme enolase complexes with RNase E (54). RNase G has also been associated with the degradation of the enolase transcript (*eno*) as well as the alcohol dehydrogenase transcript (*adhE*) (55). Other metabolic enzymes that were profoundly affected by deletion of RNase G included phosphoglucose isomerase (*pgi*), glucokinase (*glk*), and triosephosphate isomerase (*tpiA*) (56). Other links between RNA metabolism and central metabolism include the ability of citrate to bind PNPase and act as either an allosteric regulator stimulating PNPase activity or an inhibitor in binding the catalytic site, causing significant effects on central metabolism (57).

RNA sequencing has proved to be a useful tool for identifying RNase cleavage sites and can hopefully be used to better understand RNase activity and specificity so that a more thorough picture of RNA turnover can be developed. We have identified novel RNase III cleavage sites in *E. coli* and describe four modes of regulation. Two of these novel cleavage sites may affect the abundance of key metabolic enzymes, suggesting that ribonucleases may play an underappreciated role in ensuring proper regulation of central metabolic enzymes.

## MATERIALS AND METHODS

### Sample collection.

We identified cleavage sites of RNase III by collecting and sequencing RNA from both an RNase III mutant (SK4455; *rnc*-*14*::ΔTn*10 thyA715 rph-1*) (27) and the parental strain (MG1693; *thyA715 rph-1*) from which the RNase III mutant was derived (58). Samples from exponentially growing cells (lysogeny broth; 37°C with shaking) were harvested after the addition of rifampin (250 μg/ml) to stop nascent transcription and quenched with stop solution (10% phenol–ethanol). The first time point in the experiment was 3.5 min after addition of the antibiotic to give time for the diffusion of rifampin into the cells and binding of the antibiotic to RNA polymerase. This sample was called the 0-min sample, and further samples were taken from wild-type *E. coli* and the RNase III mutant at 2.5, 5, 7.5, 10, and 20 min. A partial biological replicate of the wild type was taken (at 0, 2.5, and 7.5 min) to examine the reproducibility between samples.

### RNA isolation and RNA sequencing.

Total RNA was extracted and DNase treated according to published protocols (59) and submitted to the University of Wisconsin (UW) Biotech Center, where EpiCentre Ribo-Zero Gram-negative ribosomal RNA reduction kits were used to remove rRNA from 2.5 μg of total RNA. A total of 15 libraries were prepared (TruSeq RNA Library Prep) (2 × 100 bp) and sequenced at the UW DNA sequencing facility using a HiSeq 2500 system (Illumina). For all 15 samples, there were between 14 and 23 million reads, with the average Phred score above 35. Adapters were trimmed using cutadapt, and reads were aligned to the genome (NC_000913.3) using bwa 0.7.7. SAMtools was used to index and sort aligned reads. The Integrated Genomics Viewer (IGV) was used to visualize data (28). The number of reads that aligned to each position of the genome was calculated using the count function of IGV tools with the window size set to 1. Normalization factors were calculated as the average of data from three stable genes (*ssrA*, *ssrS*, and *rnpB*) and used to normalize successive time points to the time zero sample. Bases without any aligned reads were given a pseudocount of 0.01 for the purpose of calculating the ratio of the number of reads from the *rnc* deletion mutant to the number of reads from the WT. Half-life values were calculated by fitting the equation *A* = 10^−kt^, where the read coverage at time zero had to be greater than 1 read. The half-life was calculated only when the *R*^2^ value of the regression line was greater than or equal to 0.7 using the equation *t*_1/2_ = log(0.5)/*k*.

### Expression and purification of *E. coli* RNase III.

Hexahistidine-tagged RNase III derived from *E. coli* MG1655 was expressed and purified as previously described (60). Briefly, specific primers (RNase III.F and RNase III.R) were used to amplify the coding sequence of RNase III from *Escherichia coli* MG1655 genomic DNA using Phusion High-Fidelity DNA polymerase (New England Biolabs) and the resulting fragment was cloned into a BamHI/NdeI fragment of pET28b+ using Gibson assembly (61) to generate the pET28b-His-RNase III vector for expression in BL21 cells.

For expression, colonies from a fresh plate of BL21 cells transformed with pET28b-His-*rnc* were precultured in 5 ml of LB and transferred into 250 ml of prewarmed LB medium supplemented with kanamycin (Kan) (50 mg/liter) in a 1-liter baffled flask and grown at 37°C with shaking (250 rpm). Protein expression was induced with IPTG (isopropyl-β-d-thiogalactopyranoside) (1 mM final concentration) when the cells reached an optical density (OD) at 600 nm of 0.3 to 0.4. Cells were harvested by centrifugation (6,000 rpm, 10 min, 4°C, JA.10 rotor) after ~4 h of induction, aspirated, and stored at −20°C until affinity purification was performed.

Cells were suspended in 30 ml ice-cold buffer A (500 mM NaCl, 20 mM Tris-HCl, pH 7.9)–5 mM imidazole and sonicated on a Fisher Scientific Sonic Dismembrator (model 500) for 4 min (30 s on, 40 s off; small tip, 50% power) on ice. The clarified lysate and insoluble pellet fractions were harvested following centrifugation at 6,000 × *g* (JA14.5 rotor) for 20 min at 4°C. The pellet was washed in buffer A–5 mM imidazole, solubilized in buffer A–6 M urea for 3 h at 4°C, and centrifuged at 10,000 × *g* (JA14.5 rotor) for 15 min at 4°C. The 6 M urea supernatant was applied to a 5-ml HisTrap (GE Healthcare Life Sciences) column preequilibrated in buffer A–5 mM imidazole and was then washed with 10 column volumes of buffer A–5 mM imidazole and 6 column volumes of buffer A–60 mM imidazole. The protein was eluted with buffer containing 1 M NaCl, 400 mM imidazole, and 20 mM Tris-HCl (pH 7.9) (elution fraction 1, 5 ml; elution fraction 2, 5 ml; elution fraction 3, 5 ml; elution fraction 4, 15 ml). Elution fractions 2 and 3 were pooled, placed in a 12-ml Slide-A-Lyzer cassette (Life Technologies, Inc.) (molecular weight cutoff [MWCO], 10), dialyzed against 2 liters of buffer (1 M NaCl, 400 mM imidazole, 60 mM Tris-HCl, pH 7.9) at 4°C for 2 h, and then dialyzed against 2 liters of the same buffer but without imidazole. The sample was then dialyzed against buffer (1 M NaCl, 60 mM Tris-HCl [pH 7.9], 1 mM EDTA, 1 mM dithiothreitol [DTT]) for 16 h at 4°C. The protein was concentrated (Amicon; Millipore) (MWCO, 10) to ~1.5 mg/ml as estimated by absorbance at 280 nm (ε = 14,440 M^−1^ cm^−1^). Proteins were resolved by SDS-PAGE (Any kD Mini-Protein protein gel; Bio-Rad) and stained with GelCode Blue (Themo Scientific) (see Fig. S7 in the supplemental material). The purity of the protein was estimated to be ~90% based on densitometry. The purified protein (0.77 mg/ml) was stored in aliquots at −20°C in 50% (vol/vol) glycerol–0.5 M NaCl–30 mM Tris-HCl (pH 7.9)–0.5 mM EDTA–0.5 mM DTT until use.

10.1128/mBio.00128-17.7 FIG S7  Purification of *E. coli* RNase III by immobilized metal affinity chromatography. Download FIG S7, EPS file, 1.9 MB. Copyright © 2017 Gordon et al. 2017 Gordon et al. This content is distributed under the terms of the Creative Commons Attribution 4.0 International license.

### *In vitro* RNA cleavage assays.

DNA primers were designed to amplify regions surrounding potential cleavage sites, with the forward primer containing the T7 promoter at the 5′ end of the primer. All oligonucelotides are listed in Table S2 in the supplemental material. DNA was amplified with Phusion High-Fidelity DNA polymerase (New England Biolabs), and product size was verified on a 1% DNA agarose gel. PCR products were purified by ethanol precipitation, and RNA was transcribed with a T7 RiboMAX Express large-scale RNA production system (Promega). RNA was quantified with a Qubit 2.0 Fluorometer using an RNA HS assay kit (Thermo Fisher Scientific). Five micrograms of RNA was briefly heated at 100°C for 30 s before being placed on ice. RNA was diluted in cleavage reaction buffer (30 mM Tris [pH 8], 160 mM NaCl, 0.1 mM EDTA, 0.1 mM DTT) for a total reaction volume of 100 μl. Ten microliters was removed before the addition of 0.70 μg His-RNase III and before the addition of MgCl_2_. Cleavage was initiated with addition of 10 mM (final concentration) MgCl_2_ and placed at 37°C for 15 min. Reactions were quenched by addition of EDTA at 40 mM (final concentration) in 2× urea sample loading buffer (Bio-Rad catalog no. 1610768). RNA was run on a 5% polyacrylamide 8 M urea gel and visualized with ethidium bromide (Fisher Scientific catalog no. BP1302-10).

10.1128/mBio.00128-17.9 TABLE S2  Primers used in this study. Download TABLE S2, XLSX file, 0.03 MB. Copyright © 2017 Gordon et al. 2017 Gordon et al. This content is distributed under the terms of the Creative Commons Attribution 4.0 International license.

5′ RACE was performed on 70 μl of the *in vitro* cleavage reaction mixtures. Reactions were stopped with addition of 40 mM EDTA (final concentration), and RNA was extracted with a phenol-chloroform and ethanol precipitation. cDNA was transcribed with Promega’s GoScript reverse-transcription kit (A5001) using gene-specific primers according to the manufacturer’s instructions. cDNA was purified using a QIAquick PCR purification kit (Qiagen catalog no. 28106). A 1 pM volume of PCR product was tailed with either dCTP or dGTP using terminal deoxynucleotidyl transferase (Thermo Fisher Scientific catalog no. EP0161). Products were PCR purified, and 1 μl was used as the template in a 40-μl PCR mixture using GoTaq Green master mix (Promega M712) containing the gene-specific primer and a primer designed to bind the tailed 5′ end. PCR products were purified and sequenced (Functional Biosciences, Madison, WI). Some cleavage reactions yielded a mixture of products, so these mixtures were subcloned using a pGEM-T vector system (Promega A3600).

### Pyruvate dehydrogenase assay.

Plates (LB plus 50 μg/ml thymine) inoculated with frozen glycerol stocks from SK4455 (*rnc* deletion mutant) and the parental line (WT; MG1693) were grown overnight at 37°C. Single colonies from plates were used to inoculate preculture tubes containing 5 ml of growth medium (LB plus 50 µg/ml thymine or M9 minimal medium containing 0.4% glucose, 0.2% Casamino Acids, and 50 µg/ml thymine), and the resulting cultures were grown overnight at 37°C with shaking at ~200 rpm. Overnight cultures were diluted to an OD at 600 nm of 0.05 in 50 ml prewarmed media (LB plus 50 µg/ml thymine or M9 minimal medium containing 0.4% glucose, 0.2% Casamino Acids, and 50 µg/ml thymine) in 250-ml baffled shake flasks and grown at 37°C with shaking at 250 rpm. Samples were collected (~3.3 ml/OD at 600 nm) at specific time points (log, early stationary, and late stationary growth) by centrifugation (FX6100 rotor; Beckman) (5,000 × g, 5 min, 4°C). Samples were aspirated, washed in 1 ml 25 mM phosphate buffer (pH 8.0), and centrifuged again at 5,000 × g, and the pellets were stored at −20°C.

Pyruvate dehydrogenase activity was measured in crude cellular lysates using an NADH-linked spectrophotometric assay as previously described (62). Briefly, frozen cell pellets were resuspended in 1 ml of buffer (50 mM potassium phosphate buffer [pH 8.0], 2 mM phenylmethylsulfonyl fluoride [PMSF]) on ice and then sonicated (Fisher Scientific Sonic Dismembrator Model 500) using the small tip (40% power, 1 s on, 10 s off, 1.5 min total). Clarified lysates were harvested following centrifugation at 16,100 × *g* for 15 min at 4°C (Eppendorf microcentrifuge), and the protein concentration was determined by comparison to calibrated bovine serum albumin standards (Fisher) using Bradford reagent (Sigma). For PDH activity analyses, crude extract was added to a reaction mixture (1 ml total) containing thymine pyrophosphate (0.2 mM), coenzyme A (CoA) (0.1 mM), MgCl_2_ ⋅ 6H_2_O (1 mM), dithiothreitol (0.3 mM), NAD^+^ (2.5 mM), and bovine serum albumin (100 µg/ml). The reaction was performed at room temperature (23°C) and was initiated by the addition of pyruvate (5 mM). The pyruvate-dependent reduction of NAD^+^ was monitored at 340 nm as a function of time (ε = 6,220 M^−1^ cm^−1^), and the enzyme activity is reported as micromoles of NADH produced per minute per milligram of protein.

### Accession number(s).

Raw sequencing files as well as read counts per base in each sample have been submitted to the NCBI GEO repository and can be found with GenBank accession number GSE95318.

## References

[1] Py B, Causton H, Mudd EA, Higgins CF 1994 A protein complex mediating mRNA degradation in *Escherichia coli*. Mol Microbiol 14:717–729. doi:10.1111/j.1365-2958.1994.tb01309.x. 7891559

[2] Commichau FM, Rothe FM, Herzberg C, Wagner E, Hellwig D, Lehnik-Habrink M, Hammer E, Völker U, Stülke J 2009 Novel activities of glycolytic enzymes in *Bacillus subtilis*: interactions with essential proteins involved in mRNA processing. Mol Cell Proteomics 8:1350–1360. doi:10.1074/mcp.M800546-MCP200. 19193632 PMC2690492

[3] Hui MP, Foley PL, Belasco JG 2014 Messenger RNA degradation in bacterial cells. Annu Rev Genet 48:537–559. doi:10.1146/annurev-genet-120213-092340. 25292357 PMC4431577

[4] Lee K, Zhan X, Gao J, Qiu J, Feng Y, Meganathan R, Cohen SN, Georgiou G 2003 RraA: a protein inhibitor of RNase E activity that globally modulates RNA abundance in *E. coli*. Cell 114:623–634. doi:10.1016/j.cell.2003.08.003. 13678585

[5] Kim KS, Manasherob R, Cohen SN 2008 YmdB: a stress-responsive ribonuclease-binding regulator of *E. coli* RNase III activity. Genes Dev 22:3497–3508. doi:10.1101/gad.1729508. 19141481 PMC2607070

[6] Nicholson AW 1999 Function, mechanism and regulation of bacterial ribonucleases. FEMS Microbiol Rev 23:371–390. doi:10.1111/j.1574-6976.1999.tb00405.x. 10371039

[7] Court DL 1993 RNA processing and degradation by RNase III, 71–116. *In* Belasco J, Brawerman G (ed), Control of messenger RNA stability. Academic Press, Inc., New York, NY.

[8] Schlessinger D, Ono M, Nikolaev N, Silengo L 1974 Accumulation of 30S preribosomal ribonucleic acid in an *Escherichia coli* mutant treated with chloramphenicol. Biochemistry 13:4268–4271. doi:10.1021/bi00718a004. 4606170

[9] King TC, Sirdeshmukh R, Schlessinger D 1984 RNase III cleavage is obligate for maturation but not for function of *Escherichia coli* pre-23S rRNA. Proc Natl Acad Sci U S A 81:185–188. doi:10.1073/pnas.81.1.185. 6364133 PMC344635

[10] Young RA, Steitz JA 1978 Complementary sequences 1700 nucleotides apart form a ribonuclease III cleavage site in *Escherichia coli* ribosomal precursor RNA. Proc Natl Acad Sci U S A 75:3593–3597. doi:10.1073/pnas.75.8.3593. 358189 PMC392831

[11] Court DL, Gan J, Liang YH, Shaw GX, Tropea JE, Costantino N, Waugh DS, Ji X 2013 RNase III: genetics and function; structure and mechanism. Annu Rev Genet 47:405–431. doi:10.1146/annurev-genet-110711-155618. 24274754 PMC6311387

[12] Durand S, Gilet L, Condon C 2012 The essential function of *B. subtilis* RNase III is to silence foreign toxin genes. PLoS Genet 8:e1003181. doi:10.1371/journal.pgen.1003181. 23300471 PMC3531473

[13] Matsunaga J, Simons EL, Simons RW 1996 RNase III autoregulation: structure and function of *rncO*, the posttranscriptional “operator”. RNA 2:1228–1240. 8972772 PMC1369450

[14] Bardwell JCA, Régnier P, Chen SM, Nakamura Y, Grunberg MM, Court DL 1989 Autoregulation of RNase III operon by mRNA processing. EMBO J 8:3401–3407. 2583104 10.1002/j.1460-2075.1989.tb08504.x PMC401487

[15] Régnier P, Portier C 1986 Initiation, attenuation and RNase III processing of transcripts from the *Escherichia coli* operon encoding ribosomal protein S15 and polynucleotide phosphorylase. J Mol Biol 187:23–32. doi:10.1016/0022-2836(86)90403-1. 3007765

[16] Aristarkhov A, Mikulskis A, Belasco JG, Lin ECC 1996 Translation of the adhE transcript to produce ethanol dehydrogenase requires RNase III cleavage in Escherichia coli. J Bacteriol 178:4327–4332. doi:10.1128/jb.178.14.4327-4332.1996. 8763968 PMC178197

[17] Altuvia S, Locker-Giladi H, Koby S, Ben-Nun O, Oppenheim AB 1987 RNase III stimulates the translation of the *cIII* gene of bacteriophage lambda. Proc Natl Acad Sci U S A 84:6511–6515. doi:10.1073/pnas.84.18.6511. 2957696 PMC299107

[18] Plunkett GG III, Echols H 1989 Retroregulation of the bacteriophage lambda int gene: limited secondary degradation of the RNase III-processed transcript. J Bacteriol 171:588–592. doi:10.1128/jb.171.1.588-592.1989. 2521618 PMC209629

[19] Steege DA, Cone KC, Queen C, Rosenberg M 1987 Bacteriophage lambda *N* gene leader RNA. RNA processing and translational initiation signals. J Biol Chem 262:17651–17658. 2961741

[20] Dunn JJ, Studier FW 1973 T7 early RNAs and *Escherichia coli* ribosomal RNAs are cut from large precursor RNAs *in* *vivo* by ribonuclease 3. Proc Natl Acad Sci U S A 70:3296–3300. doi:10.1073/pnas.70.12.3296. 4587248 PMC427223

[21] Majumder HK, Bishayee S, Chakraborty PR, Maitra U 1977 Ribonuclease III cleavage of bacteriophage T3RNA polymerase transcripts to late T3 mRNAs. Proc Natl Acad Sci U S A 74:4891–4894. doi:10.1073/pnas.74.11.4891. 337303 PMC432062

[22] Deltcheva E, Chylinski K, Sharma CM, Gonzales K, Chao Y, Pirzada ZA, Eckert MR, Vogel J, Charpentier E 2011 CRISPR RNA maturation by trans-encoded small RNA and host factor RNase III. Nature 471:602–607. doi:10.1038/nature09886. 21455174 PMC3070239

[23] Blomberg P, Wagner EG, Nordström K 1990 Control of replication of plasmid R1: the duplex between the antisense RNA, CopA, and its target, CopT, is processed specifically *in* *vivo* and *in vitro* by RNase III. EMBO J 9:2331–2340. 1694128 10.1002/j.1460-2075.1990.tb07405.x PMC551961

[24] Bernstein E, Caudy AA, Hammond SM, Hannon GJ 2001 Role for a bidentate ribonuclease in the initiation step of RNA interference. Nature 409:363–366. doi:10.1038/35053110. 11201747

[25] Lee Y, Ahn C, Han J, Choi H, Kim J, Yim J, Lee J, Provost P, Rådmark O, Kim S, Kim VN 2003 The nuclear RNase III Drosha initiates microRNA processing. Nature 425:415–419. doi:10.1038/nature01957. 14508493

[26] Drider D, Condon C 2004 The continuing story of endoribonuclease III. J Mol Microbiol Biotechnol 8:195–200. doi:10.1159/000086700. 16179796

[27] Stead MB, Marshburn S, Mohanty BK, Mitra J, Pena Castillo LP, Ray D, Van Bakel H, Hughes TR, Kushner SR 2011 Analysis of *Escherichia coli* RNase E and RNase III activity *in* *vivo* using tiling microarrays. Nucleic Acids Res 39:3188–3203. doi:10.1093/nar/gkq1242. 21149258 PMC3082872

[28] Robinson JT, Thorvaldsdóttir H, Winckler W, Guttman M, Lander ES, Getz G, Mesirov JP 2011 Integrative genomics viewer. Nat Biotechnol 29:24–26. doi:10.1038/nbt.1754. 21221095 PMC3346182

[29] Matsunaga J, Simons EL, Simons RW 1997 *Escherichia coli* RNase III (*rnc*) autoregulation occurs independently of *rnc* gene translation. Mol Microbiol 26:1125–1135. doi:10.1046/j.1365-2958.1997.6652007.x. 9426147

[30] Takata R, Izuhara M, Hori K 1989 Differential degradation of the *Escherichia coli* polynucleotide phosphorylase mRNA. Nucleic Acids Res 17:7441–7451. doi:10.1093/nar/17.18.7441. 2477797 PMC334822

[31] Portier C, Dondon L, Grunberg-Manago M, Régnier P 1987 The first step in the functional inactivation of the *Escherichia coli* polynucleotide phosphorylase is a ribonuclease III processing at the 5′ end. EMBO J 6:2165–2170. 3308454 10.1002/j.1460-2075.1987.tb02484.x PMC553609

[32] Lybecker M, Zimmermann B, Bilusic I, Tukhtubaeva N, Schroeder R 2014 The double-stranded transcriptome of *Escherichia coli*. Proc Natl Acad Sci U S A 111:3134–3139. 24453212 10.1073/pnas.1315974111 PMC3939876

[33] Zuker M 2003 Mfold web server for nucleic acid folding and hybridization prediction. Nucleic Acids Res 31:3406–3415. doi:10.1093/nar/gkg595. 12824337 PMC169194

[34] Lim B, Lee K 2015 Stability of the osmoregulated promoter-derived *proP* mRNA is posttranscriptionally regulated by RNase III in *Escherichia coli*. J Bacteriol 197:1297–1305. doi:10.1128/JB.02460-14. 25645556 PMC4352664

[35] Sawers G, Böck A 1989 Novel transcriptional control of the pyruvate-lyase gene: upstream regulatory sequences and multiple promoters regular anaerobic expression. J Bacteriol 171:2485–2498. doi:10.1128/jb.171.5.2485-2498.1989. 2651404 PMC209925

[36] Frunzio R, Bruni CB, Blasi F 1981 *In vivo* and *in vitro* detection of the leader RNA of the histidine operon of *Escherichia coli* K-12. Proc Natl Acad Sci USA 78:2767–2771. doi:10.1073/pnas.78.5.2767. 6166940 PMC319438

[37] Trudel M, Springer M, Graffe M, Fayat G, Blanquet S, Grunberg-Manago M 1984 Regulation of *E. coli* phenylalanyl-tRNA synthetase operon *in* *vivo*. Biochim Biophys Acta 782:10–17. 6426518 10.1016/0167-4781(84)90100-3

[38] Blasi F, Bruni CB 1981 Regulation of the histidine operon: translation-controlled transcription termination (A mechanism common to several biosynthetic operons). Curr Top Cell Regul 19:1–45. doi:10.1016/B978-0-12-152819-5.50018-X. 6277571

[39] Emory SA, Bouvet P, Belasco JG 1992 A 5′-terminal stem-loop structure can stabilize mRNA in *Escherichia coli*. Genes Dev 6:135–148. doi:10.1101/gad.6.1.135. 1370426

[40] Coburn GA, Mackie GA 1996 Overexpression, purification, and properties of *Escherichia coli* ribonuclease II. J Biol Chem 271:1048–1053. doi:10.1074/jbc.271.2.1048. 8557629

[41] Garza-Sánchez F, Schaub RE, Janssen BD, Hayes CS 2011 tmRNA regulates synthesis of the ArfA ribosome rescue factor. Mol Microbiol 80:1204–1219. doi:10.1111/j.1365-2958.2011.07638.x. 21435036 PMC3103599

[42] Lim B, Ahn S, Sim M, Lee K 2014 RNase III controls *mltD* mRNA degradation in *Escherichia coli*. Curr Microbiol 68:518–523. doi:10.1007/s00284-013-0504-5. 24343175

[43] Dip PV, Kamariah N, Subramanian Manimekalai MS, Nartey W, Balakrishna AM, Eisenhaber F, Eisenhaber B, Grüber G 2014 Structure, mechanism and ensemble formation of the alkylhydroperoxide reductase subunits AhpC and AhpF from *Escherichia coli*. Acta Crystallogr D Biol Crystallogr 70:2848–2862. doi:10.1107/S1399004714019233. 25372677

[44] Ishihama Y, Schmidt T, Rappsilber J, Mann M, Hartl FU, Kerner MJ, Frishman D 2008 Protein abundance profiling of the *Escherichia coli* cytosol. BMC Genomics 9:102. doi:10.1186/1471-2164-9-102. 18304323 PMC2292177

[45] Link AJ, Robison K, Church GM 1997 Comparing the predicted and observed properties of proteins encoded in the genome of *Escherichia coli* K-12. Electrophoresis 18:1259–1313. doi:10.1002/elps.1150180807. 9298646

[46] Régnier P, Grunberg-Manago M 1989 Cleavage by RNase III in the transcripts of the *metY-nusA-infB* operon of *Escherichia coli* releases the tRNA and initiates the decay of the downstream mRNA. J Mol Biol 210:293–302. doi:10.1016/0022-2836(89)90331-8. 2481042

[47] Olvera L, Mendoza-Vargas A, Flores N, Olvera M, Sigala JC, Gosset G, Morett E, Bolívar F 2009 Transcription analysis of central metabolism genes in *Escherichia coli*. Possible roles of σ38 in their expression, as a response to carbon limitation. PLoS One 4:e7466. 19838295 10.1371/journal.pone.0007466 PMC2759082

[48] Sauter M, Sawers RG 1990 Transcriptional analysis of the gene encoding pyruvate formate-lyase-activating enzyme of *Escherichia coli*. Mol Microbiol 4:355–363. doi:10.1111/j.1365-2958.1990.tb00603.x. 2192229

[49] Pertzev AV, Nicholson AW 2006 Characterization of RNA sequence determinants and antideterminants of processing reactivity for a minimal substrate of *Escherichia coli* ribonuclease III. Nucleic Acids Res 34:3708–3721. doi:10.1093/nar/gkl459. 16896014 PMC1540722

[50] Schmidt A, Kochanowski K, Vedelaar S, Ahrné E, Volkmer B, Callipo L, Knoops K, Bauer M, Aebersold R, Heinemann M 2016 The quantitative and condition-dependent *Escherichia coli* proteome. Nat Biotechnol 34:104–110. doi:10.1038/nbt.3418. 26641532 PMC4888949

[51] Li Z, Nimtz M, Rinas U 2014 The metabolic potential of *Escherichia coli* BL21 in defined and rich medium. Microb Cell Fact 13:45. doi:10.1186/1475-2859-13-45. 24656150 PMC4021462

[52] Tamura M, Moore CJ, Cohen SN 2013 Nutrient dependence of RNase E essentiality in *Escherichia coli*. J Bacteriol 195:1133–1141. doi:10.1128/JB.01558-12. 23275245 PMC3591997

[53] Simonte FM, Dötsch A, Galego L, Arraiano C, Gescher J 2017 Investigation on the anaerobic propionate degradation by Escherichia coli K12. Mol Microbiol 103:55–66. doi:10.1111/mmi.13541. 27671713

[54] Miczak A, Kaberdin VR, Wei CL, Lin-Chao S 1996 Proteins associated with RNase E in a multicomponent ribonucleolytic complex. Proc Natl Acad Sci U S A 93:3865–3869. doi:10.1073/pnas.93.9.3865. 8632981 PMC39450

[55] Kaga N, Umitsuki G, Nagai K, Wachi M 2002 RNase G-dependent degradation of the eno mRNA encoding a glycolysis enzyme enolase in *Escherichia coli*. Biosci Biotechnol Biochem 66:2216–2220. doi:10.1271/bbb.66.2216. 12450135

[56] Lee K, Bernstein JA, Cohen SN 2002 RNase G complementation of *rne* null mutation identifies functional interrelationships with RNase E in *Escherichia coli*. Mol Microbiol 43:1445–1456. doi:10.1046/j.1365-2958.2002.02848.x. 11952897

[57] Nurmohamed S, Vincent HA, Titman CM, Chandran V, Pears MR, Du D, Griffin JL, Callaghan AJ, Luisi BF 2011 Polynucleotide phosphorylase activity may be modulated by metabolites in *Escherichia coli*. J Biol Chem 286:14315–14323. doi:10.1074/jbc.M110.200741. 21324911 PMC3077632

[58] Bachmann BJ, Low KB 1980 Linkage map of Escherichia coli K-12, edition 6. Microbiol Rev 44:1–56. 6997720 10.1128/mr.44.1.1-56.1980 PMC373233

[59] Khodursky A, Bernstein J, Peter B, Rhodius V, Wendisch V, Zimmer D 2003 Escherichia coli spotted double-strand DNA microarrays: RNA extraction, labeling, hybridization, quality control, and data management. Methods Mol Biol 224:61–78. 12710666 10.1385/1-59259-364-X:61

[60] Amarasinghe AK, Calin-Jageman I, Harmouch A, Sun W, Nicholson AW 2001 *Escherichia coli* ribonuclease III: affinity purification of hexahistidine-tagged enzyme and assays for substrate binding and cleavage. Methods Enzymol 342:143–158. doi:10.1016/S0076-6879(01)42542-0. 11586889

[61] Gibson DG, Young L, Chuang RY, Venter JC III, Hutchison CA, Smith HO 2009 Enzymatic assembly of DNA molecules up to several hundred kilobases. Nat Methods 6:343–345. doi:10.1038/nmeth.1318. 19363495

[62] Kim Y, Ingram LO, Shanmugam KT 2008 Dihydrolipoamide dehydrogenase mutation alters the NADH sensitivity of pyruvate dehydrogenase complex of *Escherichia coli* K-12. J Bacteriol 190:3851–3858. doi:10.1128/JB.00104-08. 18375566 PMC2395023

[63] Song W, Kim YH, Sim SH, Hwang S, Lee JH, Lee Y, Bae J, Hwang J, Lee K 2014 Antibiotic stress-induced modulation of the endoribonucleolytic activity of RNase III and RNase G confers resistance to aminoglycoside antibiotics in *Escherichia coli*. Nucleic Acids Res 42:4669–4681. doi:10.1093/nar/gku093. 24489121 PMC3985665

[64] Cunningham L, Guest JR 1998 Transcription and transcript processing in the *sdh**CDAB-sucABCD* operon of *Escherichia coli*. Microbiology 144:2113–2123. doi:10.1099/00221287-144-8-2113. 9720032

[65] Barry G, Squires C, Squires CL 1980 Attenuation and processing of RNA from the *rplJL*—*rpoBC* transcription unit of *Escherichia coli*. Proc Natl Acad Sci U S A 77:3331–3335. doi:10.1073/pnas.77.6.3331. 6158044 PMC349609

[66] Downing WL, Sullivan SL, Gottesman ME, Dennis PP 1990 Sequence and transcriptional pattern of the essential *Escherichia coli* *secE-nusG* operon. J Bacteriol 172:1621–1627. doi:10.1128/jb.172.3.1621-1627.1990. 2137819 PMC208640

[67] Faubladier M, Cam K, Bouché JP 1990 *Escherichia coli* cell division inhibitor DicF-RNA of the *dicB* operon. Evidence for its generation *in* *vivo* by transcription termination and by RNase III and RNase E-dependent processing. J Mol Biol 212:461–471. doi:10.1016/0022-2836(90)90325-G. 1691299

[68] Kavalchuk K, Madhusudan S, Schnetz K 2012 RNase III initiates rapid degradation of *proU* mRNA upon hypo-osmotic stress in *Escherichia coli*. RNA Biol 9:98–109. doi:10.4161/rna.9.1.18228. 22258144

[69] Sim SH, Yeom JH, Shin C, Song WS, Shin E, Kim HM, Cha CJ, Han SH, Ha NC, Kim SW, Hahn Y, Bae J, Lee K 2010 *Escherichia coli* ribonuclease III activity is downregulated by osmotic stress: consequences for the degradation of *bdm* mRNA in biofilm formation. Mol Microbiol 75:413–425. doi:10.1111/j.1365-2958.2009.06986.x. 19943899

[70] Sim M, Lim B, Sim SH, Kim D, Jung E, Lee Y, Lee K 2014 Two tandem RNase III cleavage sites determine *betT* mRNA stability in response to osmotic stress in *Escherichia coli*. PLoS One 9:e100520. doi:10.1371/journal.pone.0100520. 24956275 PMC4067347

[71] Lim B, Sim SH, Sim M, Kim K, Jeon CO, Lee Y, Ha NC, Lee K 2012 RNase III controls the degradation of *corA* mRNA in Escherichia coli. J Bacteriol 194:2214–2220. doi:10.1128/JB.00099-12. 22343302 PMC3347049

