# Supplementary figures and images for: RNA Sequencing Identifies New RNase III Cleavage Sites in Escherichia coli and Reveals Increased Regulation of mRNA
Source: mBio. 2017 Mar 28;8(2):e00128-17. doi: 10.1128/mBio.00128-17 (PMC5371410; doi:10.1128/mBio.00128-17)

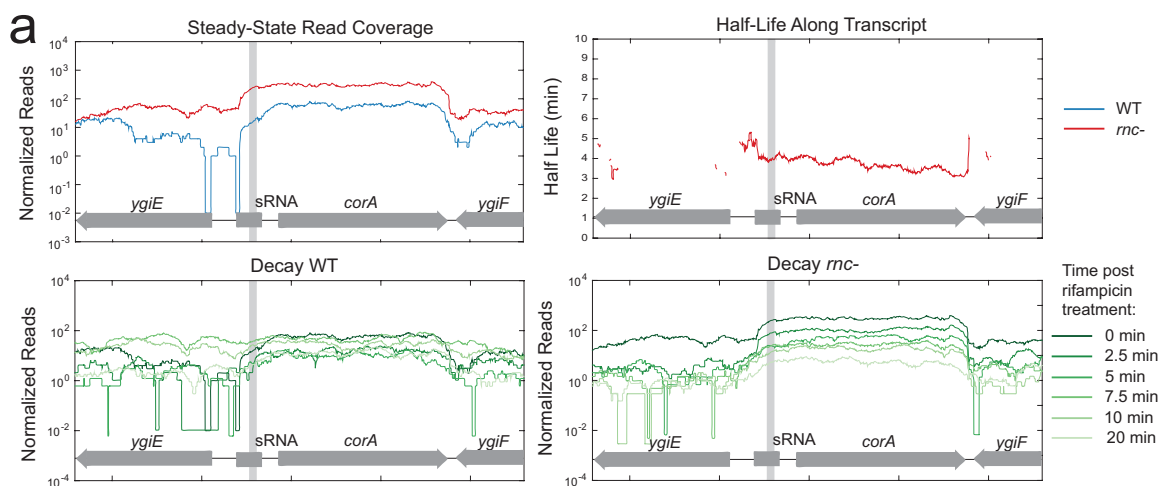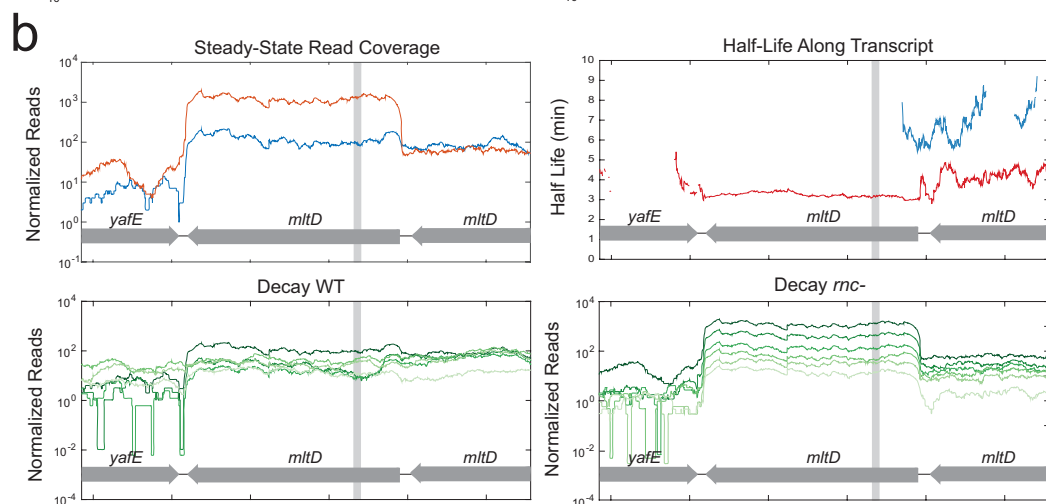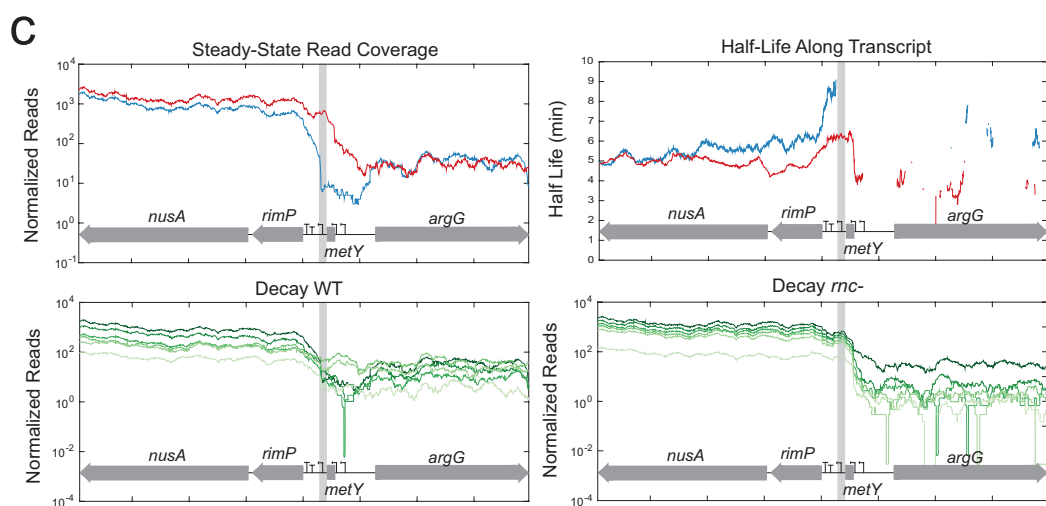

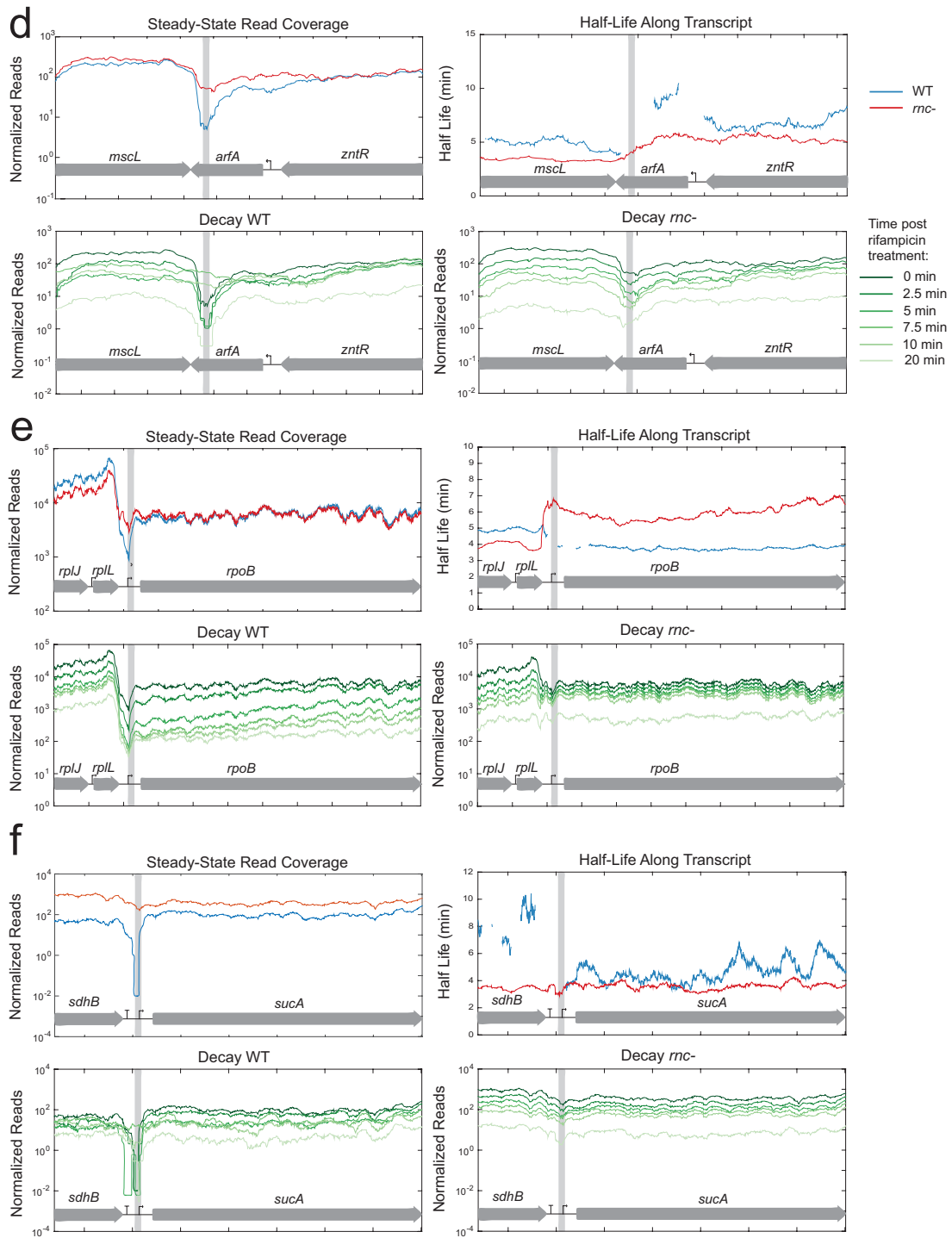

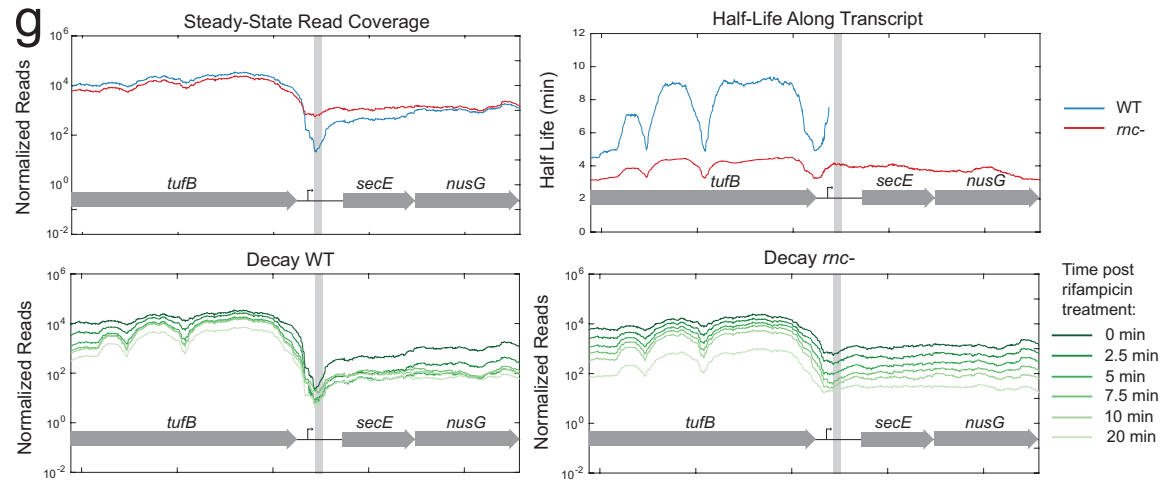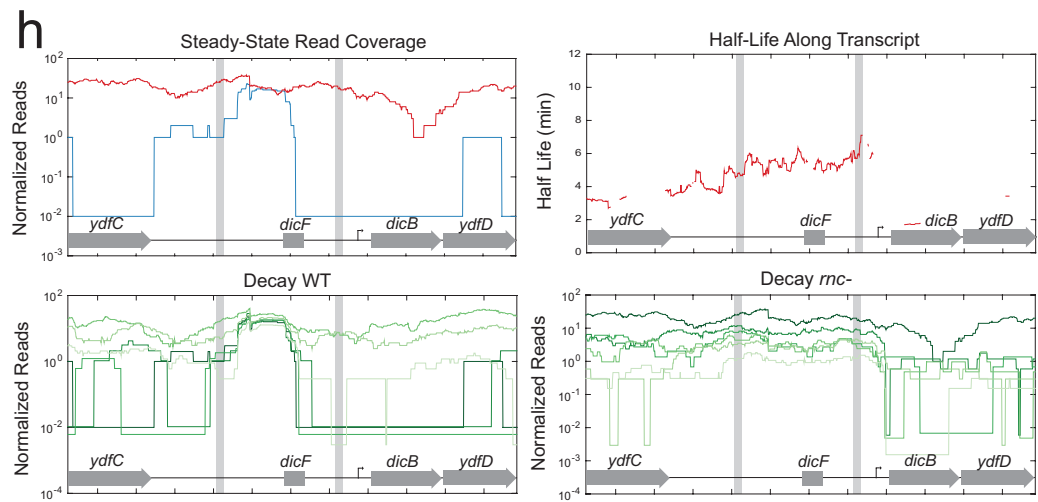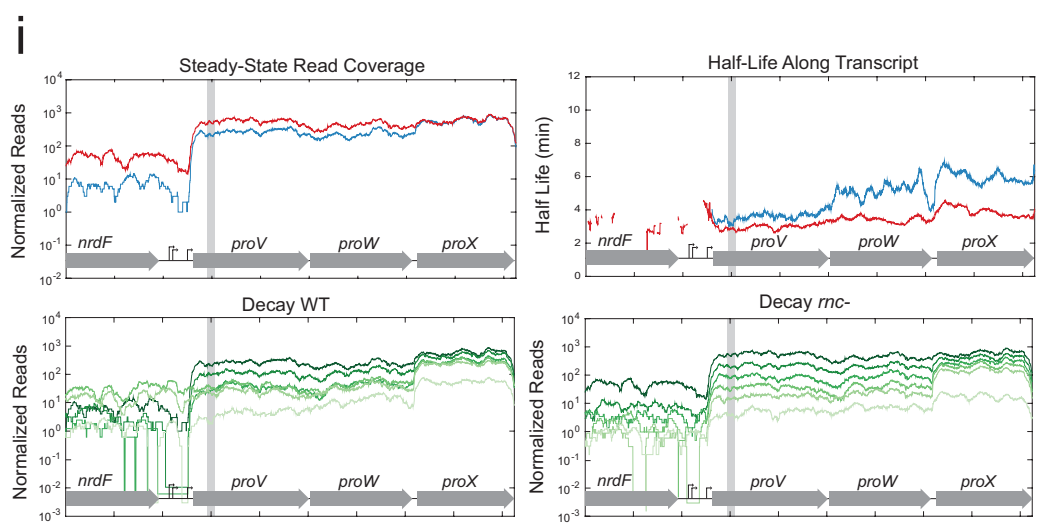

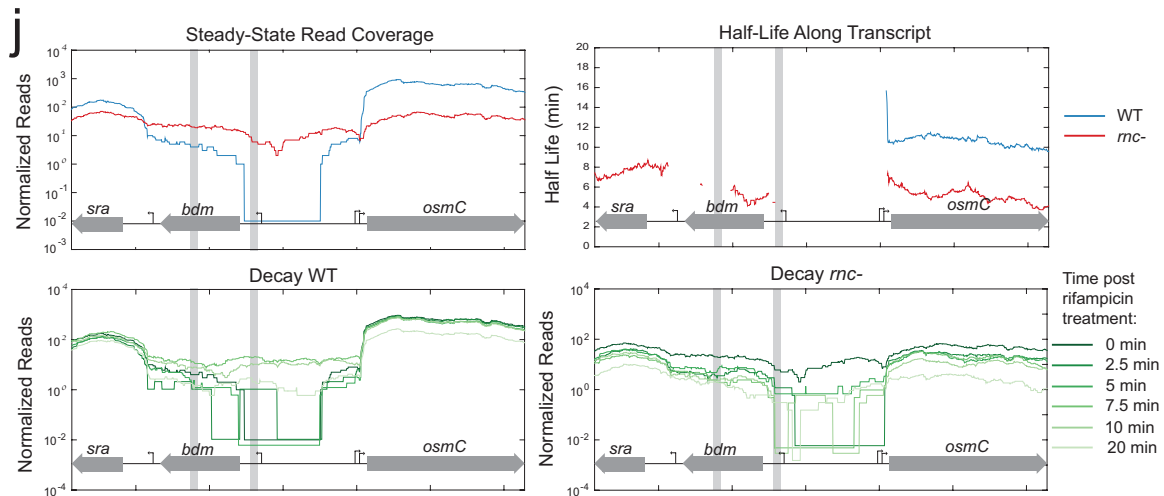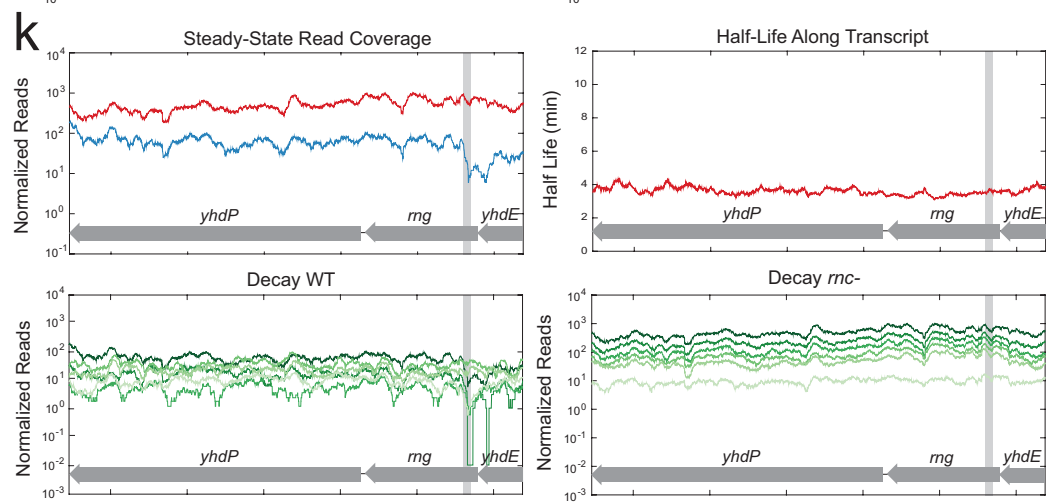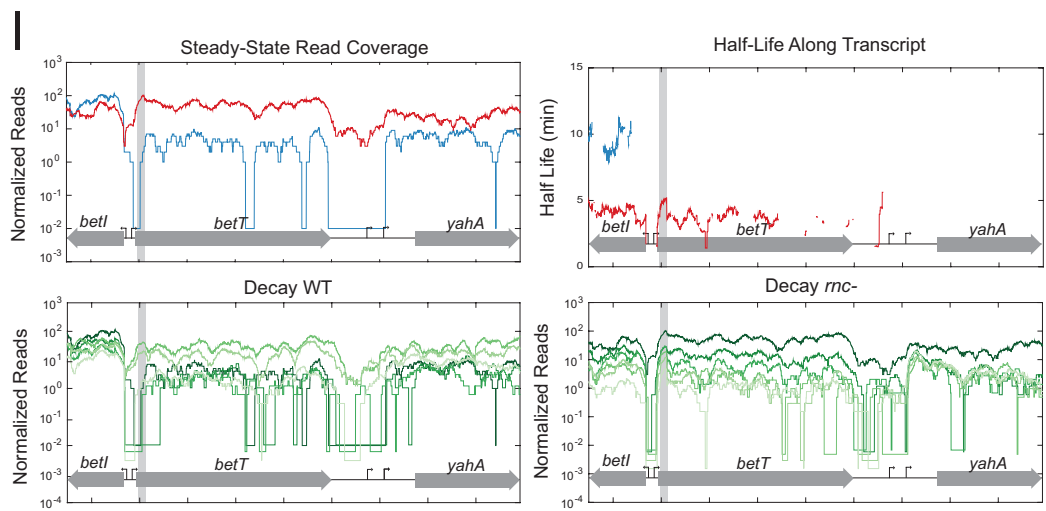

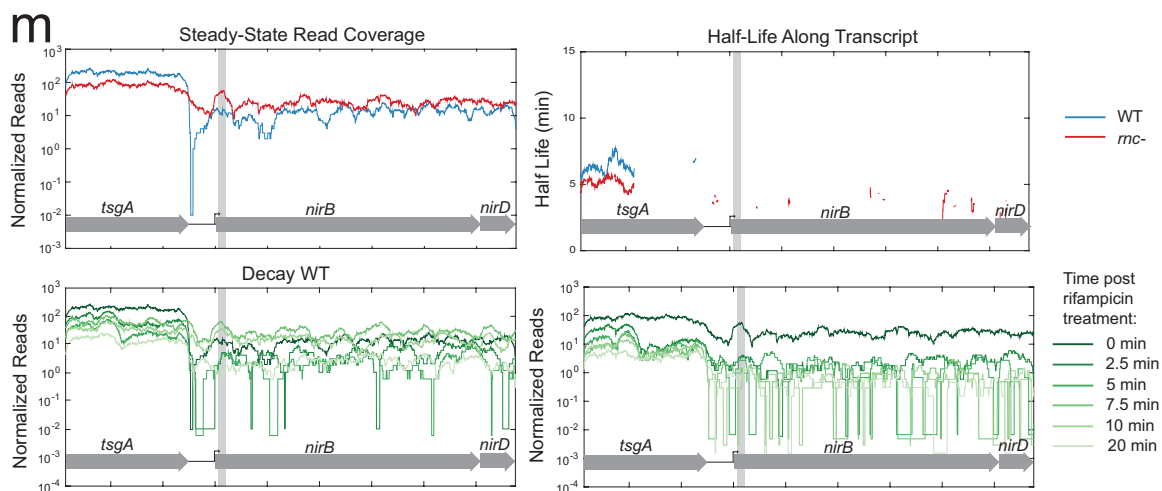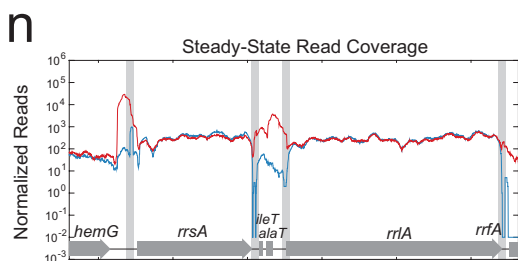

Supplement: FIG S1 [file mbo002173260sf1.pdf]

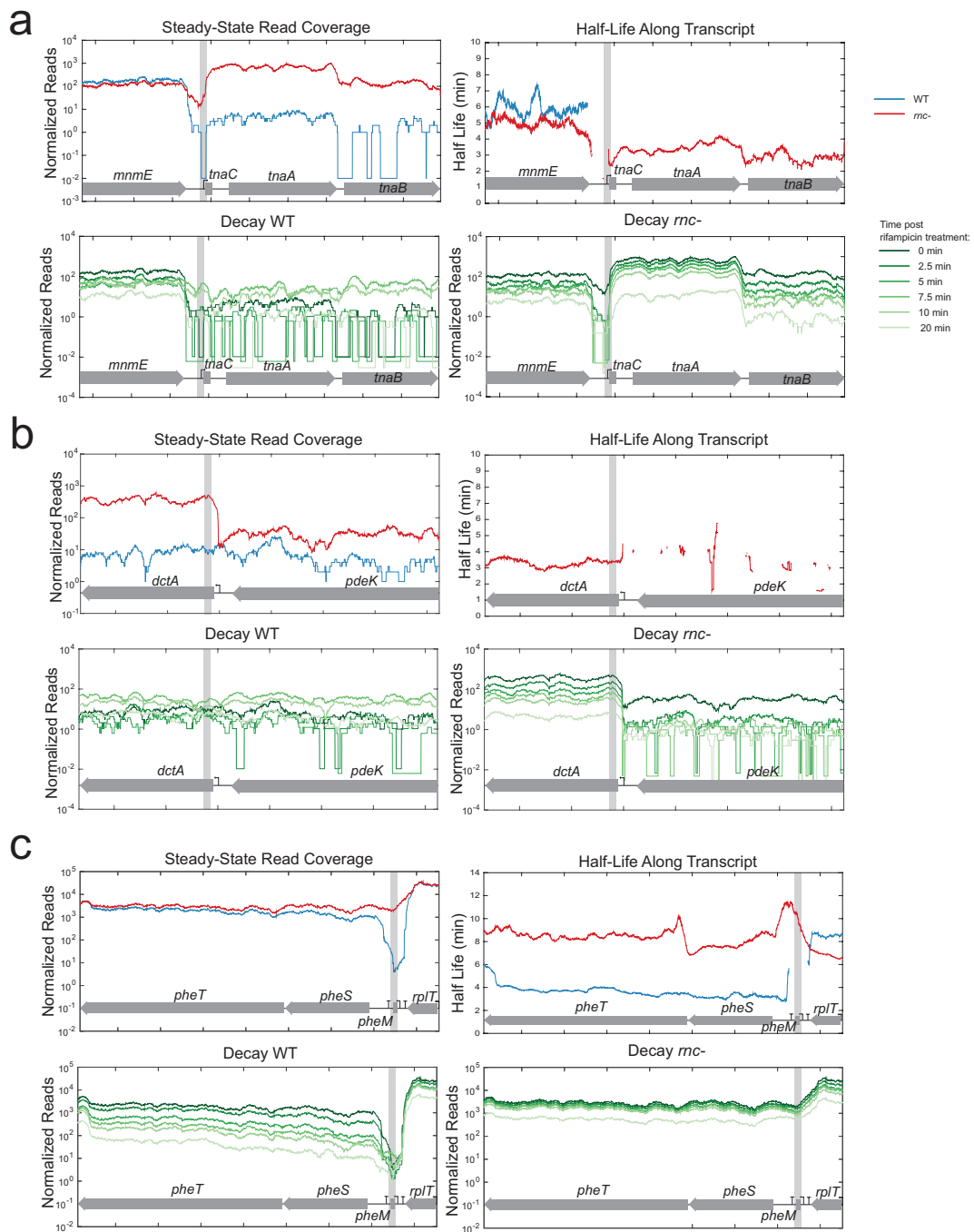

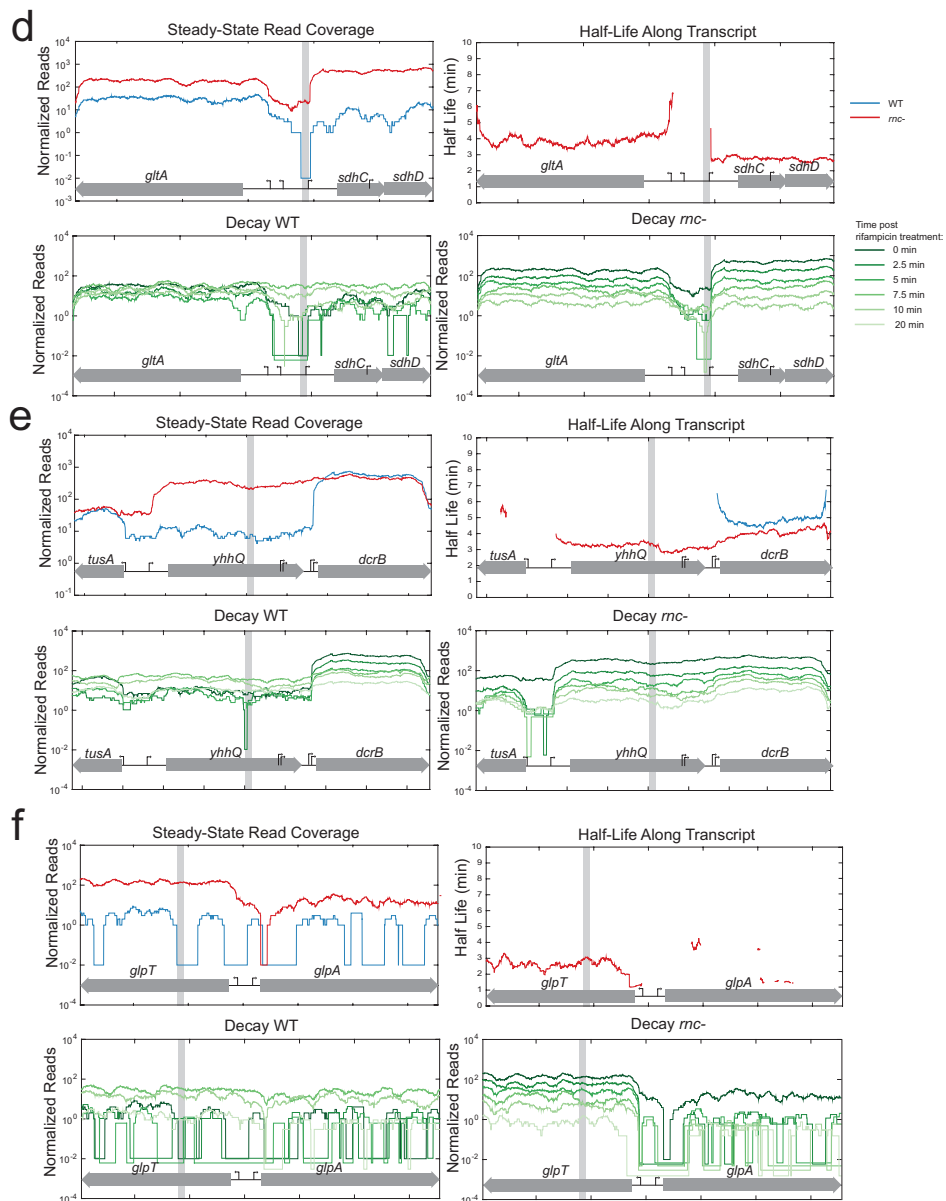

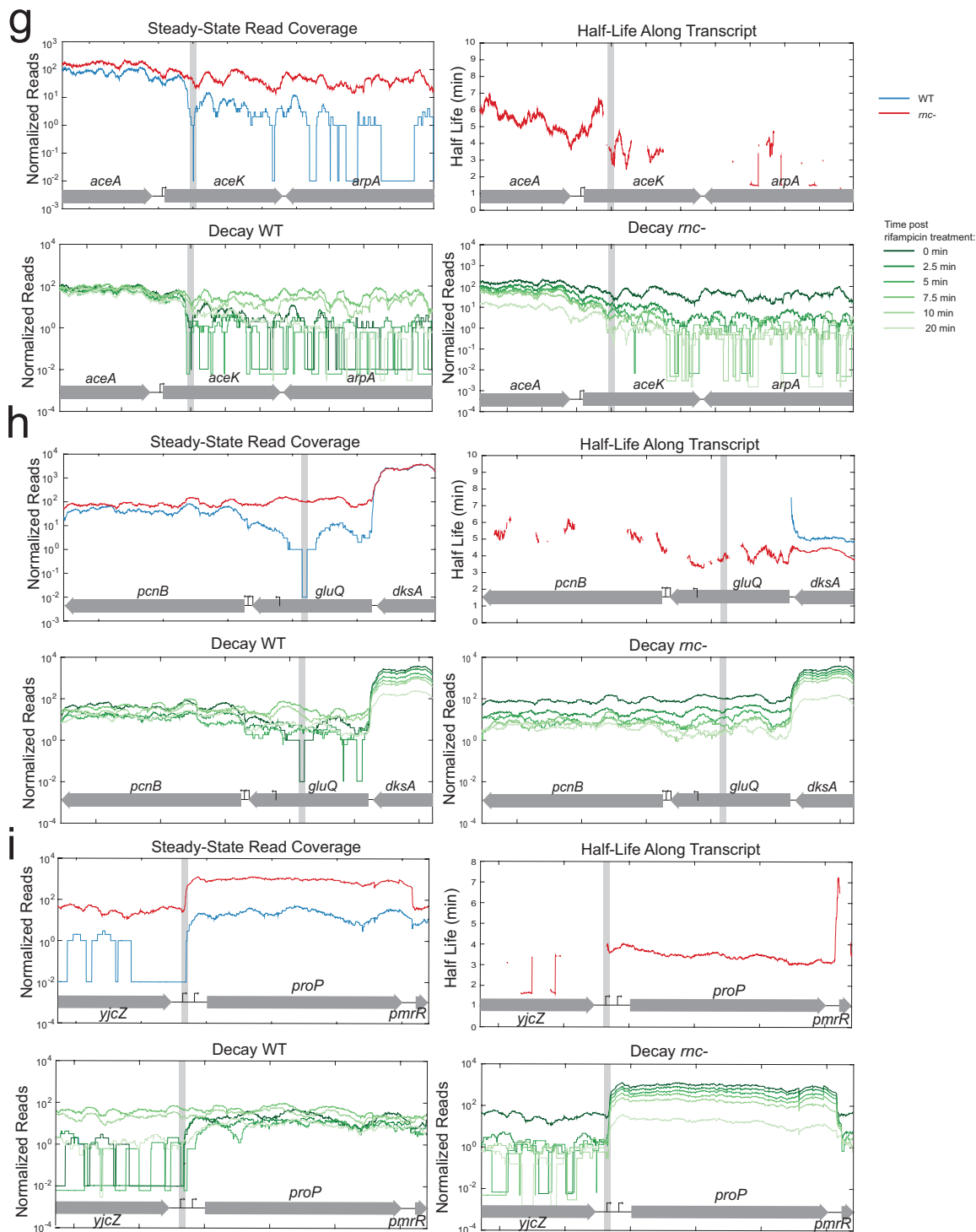

Supplement: FIG S3 [file mbo002173260sf3.pdf]
